# Site-Specific Aspartic Acid d-Isomerization in Tau R2 and R3 Peptide Seeds Attenuates Seed-Induced Fibril Formation of Full-Length Tau

**DOI:** 10.3390/biom16010143

**Published:** 2026-01-13

**Authors:** Genta Ito, Takuya Murata, Noriko Isoo, Toshihiro Hayashi, Naoko Utsunomiya-Tate

**Affiliations:** 1 Department of Biomolecular Chemistry, Faculty of Pharmaceutical Sciences, Teikyo University, Tokyo 173-8605, Japan; t-murata@pharm.teikyo-u.ac.jp; 2 Department of Physiology, School of Medicine, Teikyo University, Tokyo 173-8605, Japan; isoon@med.teikyo-u.ac.jp (N.I.); hayashi@med.teikyo-u.ac.jp (T.H.)

**Keywords:** tau, D-aspartate, isomerization

## Abstract

The aggregation of tau protein is a central pathological event in Alzheimer’s disease, and this pathology is hypothesized to spread via a prion-like mechanism driven by tau “seeds”. While aggregated tau from Alzheimer’s disease brains is known to contain age-related d-isomerized aspartic acid (d-Asp) residues, it remains unknown how this modification affects the seeding activity that drives disease propagation. Here, we investigated the impact of site-specific d-isomerization within R2 and R3 tau repeat-domain peptides, which form the core of tau fibrils. We demonstrate that the stereochemical integrity of these peptides is critical for their seeding function. d-isomerization at Asp314 within the R3 peptide seed severely impaired its ability to template the fibrillization of full-length tau in vitro. This finding was validated in a cellular model, where R3 seeds containing d-Asp314 were significantly less potent at inducing the formation of phosphorylated tau aggregates compared to wild-type seeds. Our results establish that Asp d-isomerization within tau seeds acts as a potent attenuator of their pathological seeding activity, suggesting this spontaneous modification may intrinsically modulate the progression of Alzheimer’s disease.

## 1. Introduction

Alzheimer’s disease (AD) is pathologically characterized by the accumulation of hyperphosphorylated tau proteins, which form neurofibrillary tangles (NFTs) and ultimately lead to neurodegeneration [1,2]. While extensive phosphorylation is recognized as the hallmark post-translational modification of tau in AD, the d-isomerization of aspartate (Asp) residues is also detected in NFTs [3,4,5]. This d-isomerization of Asp, known to accumulate in proteins with aging and also found in amyloid β (Aβ) peptides—another key pathogenic molecule aggregating as senile plaques in AD brains—has garnered significant attention for its potential role in AD pathogenesis [6,7].

The accumulation of NFTs in AD brains follows a stereotypical pattern, typically initiating in the entorhinal cortex and propagating into the neocortex via the hippocampal region [8,9]. This characteristic anatomical spread strongly supports the prion-like propagation hypothesis, which posits that pathological tau species are transferred between neurons, thereby inducing the aggregation of soluble tau in neighboring cells [10]. A key experimental observation supporting this model is the “seeding” effect: the addition of short tau fibrils dramatically facilitates new fibril formation. This phenomenon has been demonstrated in vitro [11], in cultured cells [12], and in vivo [13,14,15]. Therefore, it is highly probable that the inter-neuronal spread of these tau seeds is the main driving force behind tau pathology propagation in AD brains [16].

The presence of d-Asp residues in tau accumulated as paired helical filaments (PHF-tau) in AD brains was established decades ago [3,5], yet the precise localization of these modifications has remained largely obscure. While a recent proteomic study indicated Asp isomerization in AD brain tau, specifically suggesting modification at Asp387 [17,18], a comprehensive map of d-isomerization sites across the entire tau protein is still lacking. Establishing such a map requires the development of exhaustive isomeric profiling methods, which remains a significant challenge for future research. Given this current limitation, we focused on the microtubule-binding repeat domain [19,20,21], which constitutes the structural core of tau fibrils [22].

**Figure 1:**
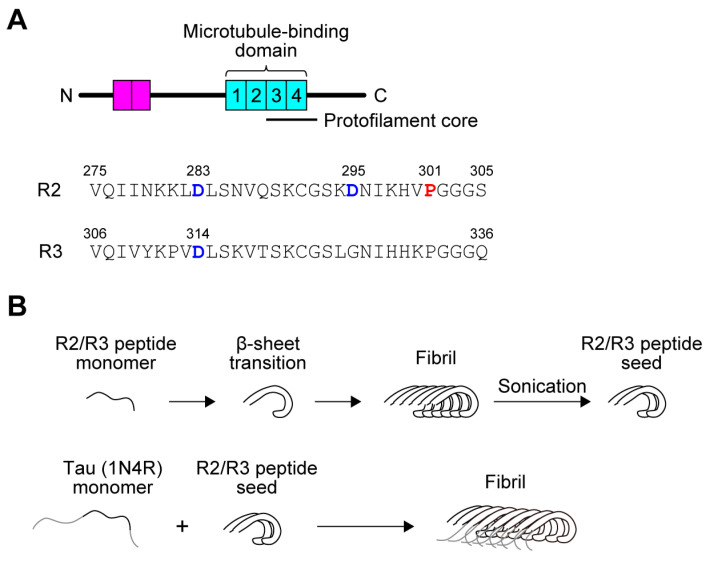
Schematics of human tau protein and its fibril formation induced by seeds. (**A**) Schematic of the human tau (2N4R) domain structure and the amino acid sequences of the R2 and R3 repeats. N-terminal insertions (2N) are shown in magenta, and C-terminal repeats (4R) are in cyan. Aspartic acid residues (blue) investigated for d-isomerization and the P301 residue linked with frontotemporal dementia (red) are highlighted. Note: All residue numbering is based on the 2N4R isoform, although full-length 1N4R tau was used in this study. (**B**) Schematic of the seed preparation and seeded fibrillization assay. (**Top**) Synthetic R2/R3 peptide monomers first undergo a structural transition to a β-sheet conformation. These intermediates subsequently polymerize into mature amyloid fibrils, which are characterized by a stable cross β-sheet structure. These pre-formed fibrils are then fragmented by sonication to generate active seeds. (**Bottom**) The peptide seeds are introduced to full-length tau (1N4R) monomers to template and accelerate their fibrillization. Regions of the full-length monomer external to the peptide core are shown in gray.

Human tau is expressed as six isoforms, formed by combinations of zero, one, or two amino-terminal insertions (0N, 1N, or 2N) and three or four carboxy-terminal repeats (3R or 4R) (Figure 1A) [23]. Based on the knowledge that the repeat domain functions as the core machinery for fibrillization, we hypothesized that spontaneous Asp isomerization within this critical region would have the most profound impact on the subsequent assembly kinetics and pathogenicity. Thus, we targeted Asp residues integral to the fibril core, specifically Asp283 and Asp295 in R2, and Asp314 in R3, as the most physiologically relevant candidate sites for investigation.

In our previous work, we demonstrated that R2 and R3 peptides derived from the repeat domain undergo a β-sheet transition, forming amyloid fibrils that bind Thioflavin T (ThT) in vitro [24]. Subsequently, we investigated how Asp d-isomerization affects the β-sheet transition, fibril formation rate, fibril morphology, and potency of tau aggregation inhibitors in R2 and R3 peptides [25]. Our specific finding was a remarkable contrast: Asp d-isomerization in R2 peptides inhibits their fibril formation, whereas the same modification in R3 peptides promotes it.

In this study, we characterized the seeding efficiency of R2 and R3 seeds on full-length tau. We demonstrated that seeds derived from both R2 and R3 peptide fibrils effectively promote the formation of full-length tau fibrils. Crucially, Asp d-isomerization in both R2 and R3 peptide seeds consistently inhibited this seeding effect in vitro. This inhibitory effect was further confirmed in cultured cells. These findings collectively suggest that Asp d-isomerization in tau seeds generally reduces their prion-like propagation ability, highlighting this modification as a potential regulatory factor in tau pathology spread.

## 2. Materials and Methods

### 2.1. Peptides and Reagents

The tau R2 and R3 peptides were custom-synthesized by Scrum, Inc. (Tokyo, Japan). Thioflavin T (ThT) dye and heparin were purchased from Fujifilm Wako Pure Chemical Corporation (Osaka, Japan). Sarkosyl (N-lauroylsarcosine sodium salt) was purchased from Tokyo Chemical Industry (Tokyo, Japan). Lipofectamine 2000 was purchased from Thermo Fisher Scientific (Waltham, MA, USA).

### 2.2. Expression and Purification of Tau (1N4R) Recombinant Protein

Rosetta2(DE3)pLysS competent cells (Merck Millipore, Burlington, MA, USA) were transformed with a pET-28a(+) plasmid (Merck Millipore) encoding full-length human tau (1N4R) wild-type (WT) or P301S, which is tagged with 6His-T7 at the N-terminus and with 6His at the C-terminus. A single colony was cultured overnight in 10 mL of LB medium containing 50 μg/mL kanamycin and 30 μg/mL chloramphenicol at 37 °C with agitation at 160 rpm. The overnight culture was diluted 100-fold in fresh medium and kept shaking at 37 °C, 160 rpm until the OD600 reached 0.4–0.8. Isopropyl-β-D-galactopyranoside was then added at a final concentration of 1 mM. The culture was cooled down to 16 °C and kept shaking at 160 rpm overnight. The bacterial culture was then centrifuged at 5000× *g* at 4 °C for 10 min. The resulting pellet was washed once with phosphate-buffered saline (PBS) and frozen at −80 °C.

The pellet was resuspended in lysis buffer (20 mM Tris-HCl pH 8.0, 500 mM NaCl, 2 mM DTT, 1 mM PMSF, complete protease inhibitor cocktail (Roche, Basel, Switzerland)) and sonicated on ice to disrupt the cells. The bacterial lysate was then centrifuged at 12,000× *g* at 4 °C for 10 min. The cleared lysate was heated at 95 °C for 10 min. The heated lysate was then centrifuged at 12,000× *g* at 4 °C for 10 min. The resulting supernatant was filtered with a 0.45 μm syringe filter. The 6His-T7-tau (1N4R)-6His protein was purified using Ni-NTA agarose (Fujifilm Wako, Osaka, Japan) and eluted with an elution buffer (20 mM Tris-HCl pH 8.0, 100 mM NaCl, 300 mM imidazole, 2 mM DTT). The eluate was subjected to cation exchange chromatography using a Mono S 10/100 GL column (Cytiva, Tokyo, Japan), and the peak fraction was dialyzed against a buffer (20 mM Tris-HCl pH 8.0, 100 mM NaCl, 1 mM DTT). Purity was confirmed by SDS-PAGE followed by Coomassie staining, and yield was quantified using a BCA protein assay kit (Takara Bio, Shiga, Japan).

### 2.3. Preparation of Tau Peptide Seeds

The tau R2 and R3 peptides were initially dissolved in 0.1% ammonium hydroxide at a concentration of 250 μM. They were diluted with 50 mM phosphate buffer (pH 7.4) to a final concentration of 30 μM and a final volume of 1 mL. Next, heparin was added to the peptide solution at a final concentration of 7.5 μM to promote fibrillization. The solutions were maintained at 37 °C without agitation for three days. The resulting fibrils were subjected to ultracentrifugation at 127,000 *g* for 20 min at 4 °C. The resulting pellet was washed once with a 30 mM Tris-HCl buffer (pH 7.5). The fibril pellet was resuspended in 100 μL of 30 mM Tris-HCl pH 7.5. The suspension was sonicated on ice to disrupt the fibrils using a hand-held sonicator. The protein concentration of the solution was quantified using a BCA protein assay kit with bovine serum albumin as the standard. The resulting seed solution was aliquoted and stored at −80 °C until use.

### 2.4. Evaluation of Seed-Induced Fibril Formation of Tau Using Thioflavin T

A reaction mixture with a total volume of 100 μL was prepared, containing the following ingredients: 20 mM Tris-HCl pH 7.5, 100 mM NaCl, 1 mM DTT, 10 μM tau (1N4R) protein, 20 μM ThT, 0.5 μM tau peptide seeds. Heparin was then added to the mixture at a final concentration of 2.5 μM to induce fibril formation. The reaction was carried out in a black 96-well plate at 37 °C. The fluorescence intensity of ThT in each well was measured every 15 min using excitation and emission wavelengths of 450 and 482 nm, respectively, on a SpectraMax i3x plate reader (Molecular Devices Japan, Tokyo, Japan) as described previously [25]. Raw fluorescence values of each replicate were normalized by the maximum value. Each replicate was analyzed separately by fitting the equation shown below on GraphPad Prism 8 (GraphPad Software Inc., Boston, MA, USA) [26].

(1) $$y=y_{i}+m_{i}x+\left(\frac{y_{f}+m_{f}x}{1+e^{-k_{app}\left(x-x_{0}\right)}}\right)$$

In this Equation (1), *y_i_* and *y_f_* are the y-intercepts of the initial (lag) and final (plateau) segments, respectively; *m_i_* and *m_f_* are the slopes of the initial and final segments, respectively; *e* is the exponential constant; *k_app_* is the apparent rate constant of the elongation segment; and *x*_0_ is the time at which the normalized ThT fluorescence value reaches 0.5. The lag time before initiating the elongation segment was calculated by subtracting 2/*k_app_* from *x*_0_. For this study, *y_i_* was set to 0 for all experiments. When the lag segment was missing, *m_i_* was also set to 0 for better fitting. All results of fitting are shown in Tables S1–S4.

### 2.5. Transmission Electron Microscopy (TEM)

TEM analysis was performed as previously described [25]. Briefly, aliquots of the tau fibril solution were adsorbed onto collodion-coated copper grids and negatively stained with 2% phosphotungstic acid. Samples were examined using a Hitachi H-7650 transmission electron microscope (Hitachi High-Technologies, Tokyo, Japan) operating at an accelerating voltage of 80 kV.

### 2.6. Seed Delivery into Neuro-2a Cells

The generation of Neuro-2a (N2a) cells that stably overexpress tau (1N4R) P301S fused with Venus at the C-terminus (tau_PS_-Venus) has been reported previously [27]. N2a cells were cultured in high-glucose Dulbecco’s modified Eagle’s medium (DMEM; Fujifilm Wako, #044-29765) supplemented with 10% (*v*/*v*) fetal bovine serum (FBS), 50 units/mL penicillin and 50 μg/mL streptomycin. The cells were cultured at 37 °C in a humidified chamber filled with 5% CO_2_/95% air. To culture N2a cells that stably overexpress tau_PS_-Venus, 0.25 μg/mL puromycin was added to the medium. Seed delivery was performed essentially as described previously [12]. For sarkosyl fractionation, cells were seeded in 6-well plates at a density of 3 × 10^5^ cells/well and cultured overnight. Mixture A was prepared by adding 50 μL of the tau seed solution to 200 μL of Opti-MEM (Thermo Fisher Scientific). Mixture B was prepared by diluting 10 μL of Lipofectamine 2000 with 240 μL of Opti-MEM. Then, mixtures A and B (final concentration of tau seed was 4 μM) were combined and incubated at room temperature for 20 min. After incubation, the mixture was added dropwise directly to the wells. For immunofluorescence, cells were seeded in 12-well plates with glass coverslips at a density of 1 × 10^5^ cells/well and cultured overnight. Seed delivery was performed as described above except that mixture A consisted of 20 μL of the tau seed solution and 80 μL of Opti-MEM, and mixture B consisted of 4 μL of Lipofectamine 2000 and 96 μL of Opti-MEM.

### 2.7. Evaluation of Tau Aggregate Formation in Neuro-2a Cells by Immunofluorescence

Seventy-two hours after seed delivery, cells were fixed with 4% (*w*/*v*) paraformaldehyde in PBS for 15 min at room temperature and washed with PBS. Cells were then permeabilized and blocked with PBS containing 0.1% (*v*/*v*) Triton X-100 and 3% (*w*/*v*) bovine serum albumin (PBS-TB) for 30 min at room temperature. Following blocking, cells were incubated overnight at 4 °C with a mouse monoclonal anti-phospho-tau (Ser202/Thr205) antibody (clone AT8; Thermo Fisher Scientific; 0.4 μg/mL) diluted in PBS-TB. After washing with PBS, cells were incubated for 1 h at room temperature in the dark with an Alexa Fluor 568-conjugated donkey anti-mouse IgG secondary antibody (A10037; Thermo Fisher Scientific; 1:500 dilution) and 300 nM DAPI for nuclear counterstaining. Coverslips were washed with PBS and mounted using ProLong Diamond Antifade Mountant (Thermo Fisher Scientific), then allowed to cure overnight at room temperature. Images were acquired using a Zeiss LSM880 confocal laser scanning microscope (Zeiss, Germany) equipped with a ×63 oil-immersion objective. Z-stack images were acquired sequentially using 405 nm (DAPI), 514 nm (Venus), 561 nm (Alexa Fluor 568) lasers (Zeiss). Image processing was performed using Image J 1.54f/Fiji (https://imagej.net/ij/index.html, last accessed on 3 December 2025), including maximum intensity projection, channel splitting, linear contrast adjustment, and merging. Contrast settings were applied identically across all samples.

### 2.8. Evaluation of Tau Aggregate Formation in Neuro-2a Cells by Sarkosyl Fractionation

Sarkosyl fractionation was performed as previously described [28]. Seventy-two hours after seed delivery, the cells were washed once with PBS and lysed in 1 mL of lysis buffer (10 mM Tris-HCl pH 7.5, 10% (*w*/*v*) sucrose, 0.8 M NaCl, 1 mM EGTA, 1% (*w*/*v*) N-lauroylsarcosine, Complete, PhosSTOP phosphatase inhibitor cocktail (Roche)). The cell lysates were harvested and incubated for 30 min on ice. The lysates were sonicated and then incubated at room temperature for 15 min. The sonicated lysates were centrifuged at 12,000× *g* for 10 min at 4 °C. The resulting supernatants were ultracentrifuged at 260,000× *g* for 20 min at 4 °C. The resulting supernatants were mixed with an equal volume of 2× Laemmli’s SDS-PAGE sample buffer for immunoblotting (sarkosyl-soluble fraction). The pellets were resuspended with 1 mL of PBS and ultracentrifugation at 260,000× *g* for 20 min at 4 °C. The pellets were then resuspended with 50 μL of 1× Laemmli’s SDS-PAGE sample buffer (sarkosyl insoluble fraction). Both sarkosyl soluble and insoluble fractions were heated to 95 °C for 5 min. Then, 5 μL of the soluble fraction and 10 μL of the insoluble fraction were loaded onto Tris-glycine 7.5% SDS-PAGE gels. Immunoblotting was performed as previously described [29]. A rat monoclonal antibody against the tau repeat domain (A16040D; BioLegend, San Diego, CA, USA) was used at a final concentration of 1 μg/mL, and a rabbit monoclonal antibody against phospho-tau (Ser202/Thr205) (#30505; Cell Signaling Technology, Danvers, MA) was used at a dilution of 1:1000. The immunoblots were developed using Amersham Imager 680 (Cytiva) and ImmunoStar Zeta (Fujifilm Wako) as a substrate. A common reference sample (S) was run alongside the experimental samples in each experiment. Quantified band intensities were first normalized to the intensity of S. Subsequently, the S-normalized intensity of insoluble phosphorylated tau was normalized to that of soluble total tau. Image J/Fiji version 1.54f (https://imagej.net/ij/index.html, last accessed on 3 December 2025) was used for quantifying band intensities. Western blot original images can be found in the Supplementary Materials.

## 3. Results

### 3.1. Aspartic Acid d-Isomerization Impairs the Seeding Activity of Tau Repeat Peptides

The d-isomerization of Asp is a hallmark of aging-associated protein aggregates, yet its precise role in seeding pathology remains unclear. We therefore aimed to elucidate how d-isomerization of Asp in tau seeds influences the fibrillization of full-length tau. To circumvent the technical challenge of producing full-length recombinant tau containing site-specific d-Asp, we utilized R2 and R3 peptides derived from the microtubule-binding domain, which readily form amyloid fibrils in vitro. The R2 peptide contains two Asp residues (Asp283, Asp295) and the R3 peptide contains one (Asp314) (Figure 1A). Seeds were subsequently prepared by sonicating pre-formed fibrils assembled from either wild-type (WT) peptides or peptides containing d-Asp residues (Figure 1B).

**Figure 2:**
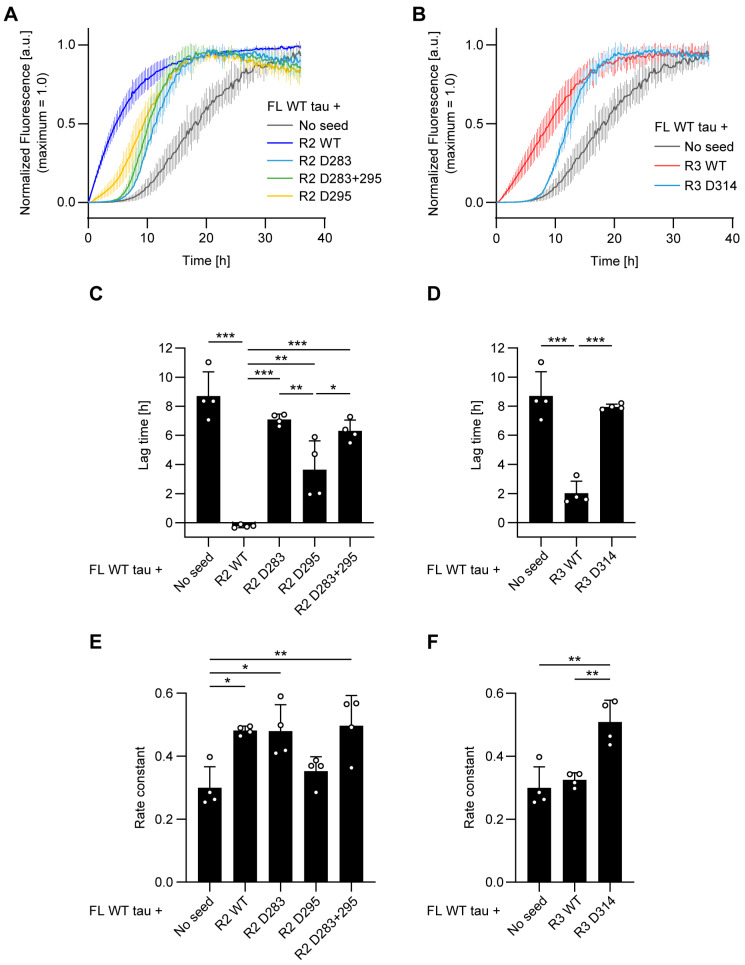
d-Isomerization of Asp in R2/R3 seeds impairs in vitro seeding of full-length WT tau. (**A**,**B**) Fibrillization kinetics of full-length (FL) WT tau, monitored by Thioflavin T (ThT) fluorescence. Reactions were conducted in the absence (no seed) or presence of the indicated R2 (**A**) or R3 (**B**) peptide seeds. Curves show the mean ± SD of normalized fluorescence from four independent experiments (n = 4). (**C**–**F**) Kinetic parameters derived from sigmoidal fitting of the data in (**A**,**B**). Bar graphs display the (**C**,**D**) lag time and (**E**,**F**) apparent rate constant. Bars represent the mean, error bars are the SD, and open circles show the individual values from each of the four experiments. The “no seed” control data are duplicated in all panels for clarity. Statistical significance was determined by one-way ANOVA with Tukey’s post hoc test: * *p* < 0.05, ** *p* < 0.01, *** *p* < 0.001. See also Figures S1–S8 and Tables S1 and S2.

We first monitored the fibril formation of full-length WT tau (1N4R) using ThT fluorescence to determine the lag time and rate constant of fibril formation. In the absence of seeds, the lag time for full-length tau fibrillization was approximately 9 h (Figure 2A,C). The addition of R2 WT seeds dramatically shortened this lag time. While R2 seeds containing d-Asp at position 283 (R2 D283), 295 (R2 D295), or both (R2 D283 + 295) also reduced the lag time, their seeding efficacy was significantly weaker than that of R2 WT seeds (Figure 2A,C). Similarly, R3 WT seeds significantly shortened the lag time to approximately 2 h, whereas R3 seeds with d-Asp at position 314 (R3 D314) failed to do so (Figure 2B,D). Interestingly, the fibril elongation rate constants were affected in a complex, position-dependent manner.

The addition of R2 WT, D283, and D283 + 295 seeds significantly increased the rate constant, whereas the R2 D295 seed did not (Figure 2E). Conversely, the rate constant was unchanged by R3 WT seeds but was significantly increased by R3 D314 seeds (Figure 2F). These results indicate that d-isomerization of Asp residues within seed peptides primarily impairs their ability to shorten the lag time of tau fibrillization, while their effects on the elongation rate are divergent and position-specific.

**Figure 3:**
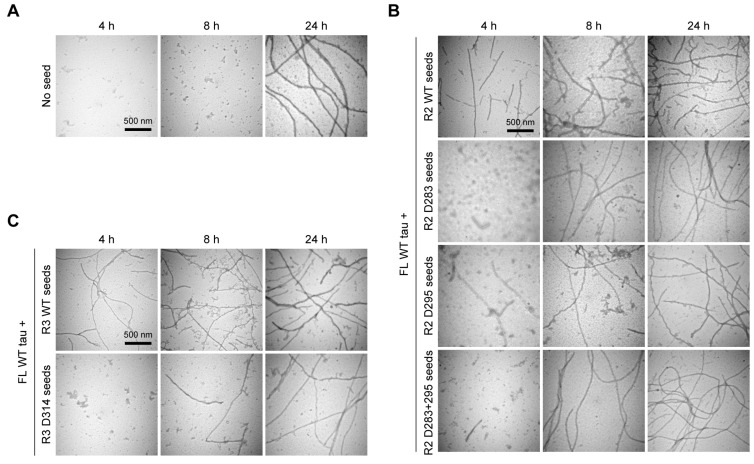
TEM images of tau WT fibrils formed in the presence of R2/R3 seeds. Transmission electron microscopy (TEM) images show the fibrillization of full-length wild-type (FL WT) tau monomers. Samples were taken 4, 8, and 24 h after initiating aggregation without seeds (**A**) or in the presence of various R2 seeds (**B**) or R3 seeds (**C**). Scale bars: 500 nm.

To confirm these kinetic findings morphologically, we used transmission electron microscopy (TEM) to visualize fibril formation at 4, 8, and 24 h (Figure 3). In unseeded reactions, mature fibrils were abundant only at 24 h, consistent with the long lag time observed by ThT (Figure 3A). In contrast, R3 WT seeds induced the formation of numerous fibrils as early as 4 h (Figure 3C, top row). R2 WT seeds also showed potent activity, with fibrils apparent by 4 h and abundant by 8 h (Figure 3B, top row). Conversely, all d-Asp-containing seeds showed delayed fibril appearance compared to WT seeds (Figure 3B,C, lower rows). These morphological data strongly correlate with the lag times calculated from the ThT assays, confirming that d-isomerization primarily impairs the initiation of tau fibrillization.

### 3.2. d-Isomerization of Asp314 Attenuates the In Vitro Seeding Activity of R3 Peptides for P301S Tau

**Figure 4:**
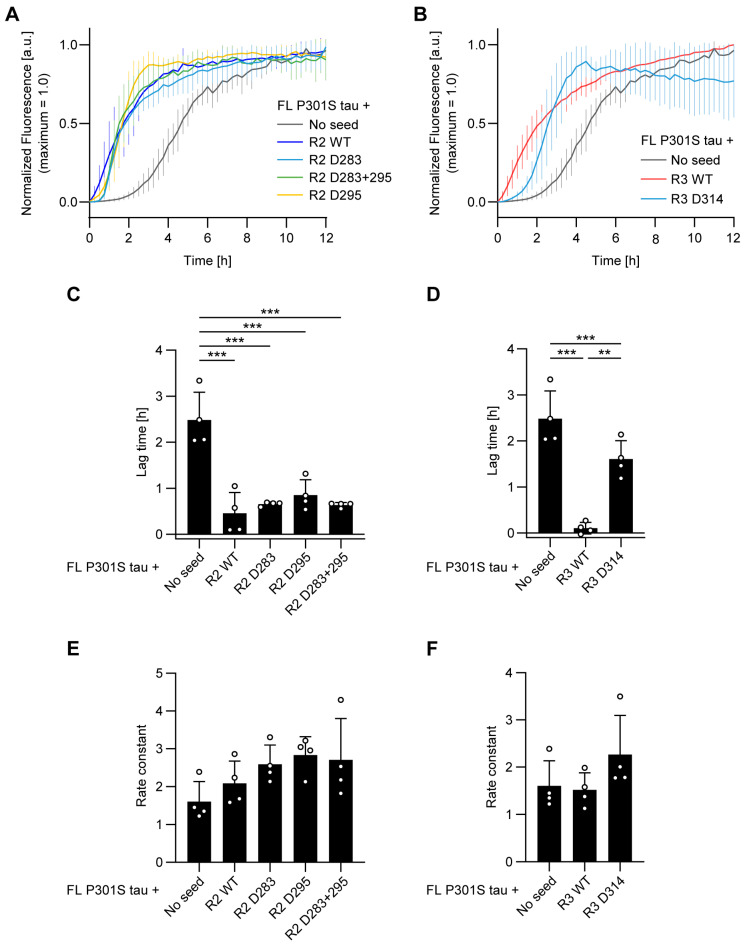
d-isomerization of Asp314, but not Asp residues in R2, attenuates the in vitro seeding of P301S tau. (**A**,**B**) Fibrillization kinetics of full-length (FL) P301S tau, monitored by Thioflavin T (ThT) fluorescence. Reactions were conducted in the absence (no seed) or presence of the indicated R2 (**A**) or R3 (**B**) peptide seeds. Curves show the mean ± SD of normalized fluorescence from four independent experiments (n = 4). (**C**–**F**) Kinetic parameters derived from sigmoidal fitting of the data in (**A**,**B**). Bar graphs display the (**C**,**D**) lag time and (**E**,**F**) apparent rate constant. Bars represent the mean, error bars are the SD, and open circles show the individual values from each of the four experiments. The “no seed” control data are duplicated in all panels for clarity. Statistical significance was determined by one-way ANOVA with Tukey’s post hoc test: ** *p* < 0.01, *** *p* < 0.001. See also Figures S9–S16 and Tables S3 and S4.

The P301S mutation in the *MAPT* gene, which is linked to inherited frontotemporal dementia, is known to accelerate tau fibrillization [30,31,32]. Given that Pro301 is located within the R2 peptide region, we investigated the effect of Asp d-isomerization in the R2/R3 seeds on the fibrillization of full-length P301S tau. Spontaneous fibrillization of P301S tau was rapid, with a lag time of approximately 2.5 h (Figure 4A,C). The addition of R2 seeds dramatically shortened the lag time, an effect that was independent of Asp isomerization within the seeds (Figure 4A,C). Similarly, R3 WT seeds markedly reduced the lag time; in contrast, the seeding effect of R3 D314 seeds was significant but less pronounced than that of the R3 WT seeds (Figure 4B,D). The fibrillization rate, however, was not altered by the addition of any of the R2 or R3 seeds (Figure 4E,F). Taken together, these results suggest that while Asp d-isomerization in R3 seeds attenuates their seeding activity for P301S tau, Asp d-isomerization in R2 seeds has no apparent effect on the seeding activity of the R2 seeds for P301S tau.

### 3.3. Cellular Seeding Activity of R3 Peptides Is Impaired by d-Isomerization at Asp314

**Figure 5:**
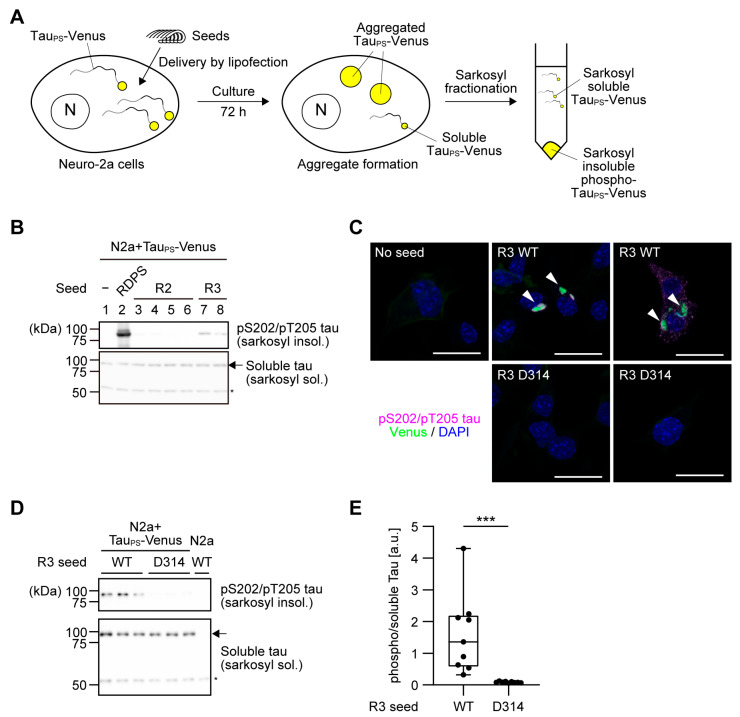
d-isomerization of Asp314 impairs R3-mediated tau seeding in a cellular model. (**A**) Schematic overview of the cellular seeding assay. R2 or R3 seeds were delivered into Neuro-2a (N2a) cells stably expressing P301S-Venus tau (tau_PS_-Venus) by lipofection. After 72 h, cells were lysed and fractionated using sarkosyl to separate soluble tau (supernatant) from sarkosyl-insoluble aggregates (pellet). (**B**) Comparison of seeding activity between R2 and R3 peptides. Representative immunoblots of the sarkosyl-insoluble fraction (**top**) and soluble fraction (**bottom**) are shown. Lane 1 is a no-seed control, whereas RDPS (seeds prepared from the entire repeat domain harboring the P301S mutation) serves as a positive control (lane 2). The arrow indicates soluble tau_PS_-Venus, and the asterisk indicates a non-specific band serving as a loading control. Lane 3: R2 WT; lane 4: R2 D283; lane 5: R2 D295; lane 6: R2 D283 + 295; lane 7: R3 WT; lane 8: R3 D314. (**C**) Representative immunofluorescence images of tau_PS_-Venus inclusions formed by the delivery of R3 WT seeds (arrowheads). Cells were stained with an anti-pS202/pT205 tau antibody (magenta). The Venus fluorescence and DAPI-stained nuclei are shown in green and blue, respectively. Scale bar: 20 μm. (**D**) Representative immunoblots showing the effect of Asp314 d-isomerization. Sarkosyl-insoluble (**top**) and soluble (**bottom**) fractions from cells treated with R3 WT or R3 D314 seeds were analyzed. The arrow indicates soluble tau_PS_-Venus. Western blot original images can be found in Figure S19. (**E**) Quantification of the sarkosyl-insoluble pS202/pT205 tau signal shown in (**D**), normalized to the corresponding soluble tau signal. Box-and-whisker plots display the data distribution from three independent experiments, each performed with three biological replicates. Individual data points are shown as dots. Statistical analysis was performed using a Mann–Whitney test (*** *p* < 0.001).

To validate our in vitro findings, we next examined the effect of Asp314 d-isomerization in R3 seeds on the aggregate formation of full-length P301S tau in a cellular model. We used murine neuroblastoma (Neuro-2a) cells stably expressing C-terminally Venus-tagged P301S tau. Following delivery with tau seeds, cellular aggregates were isolated by sarkosyl extraction and detected by immunoblotting for phosphorylated tau (Figure 5A). While R2 seeds failed to induce tau aggregation in cells (Figure 5B), delivery with R3 WT seeds resulted in formation of sarkosyl-insoluble species positive for phospho-Ser202/Thr205, albeit in smaller amounts compared with the authentic positive control seeds (repeat domain with the P301S mutation) (Figure 5B). We confirmed that inclusions positive for phospho-tau were formed upon delivery of R3 WT seeds, but not with R3 D314 seeds (Figure 5C). In cells treated with R3 D314 seeds, the levels of the sarkosyl-insoluble phospho-tau were significantly reduced (Figure 5D,E). This result indicates that d-isomerization at Asp314 attenuates the seeding activity of R3 seeds in cells, consistent with our in vitro observations.

## 4. Discussion

In this study, we demonstrated that the d-isomerization of specific Asp within tau repeat peptides potently impairs their pathological seeding activity in a position-dependent manner. The native l-configuration of Asp, particularly at position 314 in the R3 repeat, appears essential for an efficient seed template (Figure 2). Our data are consistent with the hypothesis that the stereochemical inversion from l- to d-Asp introduces a structural incompatibility at the seed–monomer interface, hindering the efficient recruitment of native tau monomers. This interpretation is supported by our kinetic data, where R3 D314 seeds completely failed to shorten the lag time of WT tau aggregation. Furthermore, our data revealed a striking “decoupling” of the fibril formation phases: the same R3 D314 seed that failed to act as an efficient template paradoxically accelerated the fibril elongation rate. This increase in the rate constant could imply that d-isomerized seeds shift the dynamic instability of the seed-monomer complex towards fibril formation, actively promoting the elongation phase. However, given that R3 D314 seeds failed to induce aggregation in cells (Figure 5), this enhanced elongation appears insufficient to overcome the primary defect in seeding nucleation, reinforcing the view that d-isomerization acts as a net inhibitor of tau pathology.

The functional consequences of this d-isomerization were revealed to be highly context-dependent. The pathogenic P301S mutation, known for its high intrinsic fibrillization propensity, completely masked the seeding deficiency of d-isomerized R2 peptides (Figure 4A,C,E). This suggests that a monomer with a high propensity for fibrillization may be less dependent on a perfectly complementary seed template. However, even this aggressive mutant could not fully compensate for the structural defect of the R3 D314 seed. While the P301S mutation enabled R3 D314 seeds to exhibit significant seeding activity—unlike their complete inactivity against WT tau—their efficiency remained substantially lower than that of R3 WT seeds (Figure 4B,D). This finding establishes a clear hierarchy: the structural integrity of Asp314, located within the fibril core, remains a critical factor for templating that was not fully overridden by the P301S mutation in our assay.

Our findings, validated in a cellular model, offer a new perspective on the role of d-isomerization in sporadic AD. The pronounced failure of d-Asp314 seeds to induce aggregation in cells, despite eventually forming fibrils in vitro, highlights a key difference between these systems. This discrepancy can be attributed to cellular protein quality control mechanisms, which are known to clear aggregating or misfolded protein species, although further research is needed to elucidate the precise interplay between d-isomerized seeds and these clearance pathways.

Regarding the origin of d-Asp incorporation, experimental evidence points to spontaneous chemical racemization rather than biological incorporation from external sources [33,34]. Asp residues are chemically prone to forming succinimide intermediates, which spontaneously hydrolyze to generate d-isomers [35]. Under normal physiological conditions, such damaged proteins are recognized and eliminated by cellular quality control systems, such as the protein l-isoaspartyl methyltransferase (PIMT) repair enzyme or specific degradation pathways [36]. Notably, we recently reported the activity of a d-aspartyl endopeptidase capable of specifically degrading d-Asp-containing peptides [37]. In the aging brain, the impairment of these specific clearance mechanisms could lead to the accumulation of soluble d-isomerized tau, potentially triggering the initial aggregation of tau. Intriguingly, however, our current study demonstrates that these d-isomerized species act as poor seeds for propagation. This suggests the existence of a paradoxical homeostatic balance in tau pathology: while the failure of quality control allows d-isomerization to drive local aggregation, the same modification intrinsically attenuates the prion-like spread of the pathology to neighboring cells. Future studies should aim to elucidate the precise molecular link between the breakdown of these specific repair/degradation machineries and the initiation of tau aggregation in the aging brain.

## 5. Conclusions

Accumulation of d-Asp is a hallmark of protein aging, observed in AD brains. While its precise role remains ambiguous, our data point toward a complex function. It is plausible that the spontaneous accumulation of d-Asp in tau seeds creates structurally defective templates, thereby reducing the efficiency of pathological propagation in the aging brain. In conclusion, this work establishes protein stereochemistry as a novel, critical factor in regulating tau propagation, linking the chemical process of aging directly to the molecular mechanism of tau seeding.

## Data Availability

The original contributions presented in this study are included in the article/Supplementary Material. Further inquiries can be directed to the corresponding author.

## Supplementary Materials

The following supporting information can be downloaded at https://www.mdpi.com/article/10.3390/biom16010143/s1, Figure S1: Individual curves corresponding to Figure 2A,B. Figure S2: Individual curve fits for WT tau (1N4R) fibril formation without seeds. Figure S3: Individual curve fits for WT tau (1N4R) fibril formation with R2 WT seeds. Figure S4: Individual curve fits for WT tau (1N4R) fibril formation with R2 D283 seeds. Figure S5: Individual curve fits for WT tau (1N4R) fibril formation with R2 D295 seeds. Figure S6: Individual curve fits for WT tau (1N4R) fibril formation with R2 D283 + 295 seeds. Figure S7: Individual curve fits for WT tau (1N4R) fibril formation with R3 WT seeds. Figure S8: Individual curve fits for WT tau (1N4R) fibril formation with R3 D314 seeds. Figure S9: Individual curves corresponding to Figure 4A,B. Figure S10: Individual curve fits for P301S tau (1N4R) fibril formation without seeds. Figure S11: Individual curve fits for P301S tau (1N4R) fibril formation with R2 WT seeds. Figure S12: Individual curve fits for P301S tau (1N4R) fibril formation with R2 D283 seeds. Figure S13: Individual curve fits for P301S tau (1N4R) fibril formation with R2 D295 seeds. Figure S14: Individual curve fits for P301S tau (1N4R) fibril formation with R2 D283 + 295 seeds. Figure S15: Individual curve fits for P301S tau (1N4R) fibril formation with R3 WT seeds. Figure S16: Individual curve fits for P301S tau (1N4R) fibril formation with R3 D314 seeds. Figure S17: Original uncropped images for Figure 5B. Figure S18: Individual channel images for Figure 5C. Figure S19: Original uncropped images for Figure 5D. Table S1: Results of curve fitting of tau (1N4R) WT fibril formation. Table S2: Parameters of tau (1N4R) WT fibril formation calculated by curve fitting. Table S3: Results of curve fitting of tau (1N4R) P301S fibril formation. Table S4: Parameters of tau (1N4R) P301S fibril formation calculated by curve fitting.

## Abbreviations

The following abbreviations are used in this manuscript: Aβ: Amyloid beta; AD: Alzheimer’s disease; ANOVA: Analysis of variance; Asp: Aspartic acid; BCA: Bicinchoninic acid; d-Asp: d-isomerized aspartic acid; DMEM: Dulbecco’s modified Eagle’s medium; DTT: Dithiothreitol; EGTA: Ethylene glycol-bis(beta-aminoethyl ether)-N,N,N′,N′-tetraacetic acid; FBS: Fetal bovine serum; FL: Full-length; LB: Luria–Bertani; MAPT: Microtubule-associated protein tau; N2a: Neuro-2a; NFTs: Neurofibrillary tangles; OD: Optical density; PBS: Phosphate-buffered saline; PMSF: Phenylmethylsulfonyl fluoride; SD: Standard deviation; SDS-PAGE: Sodium dodecyl sulfate-polyacrylamide gel electrophoresis; TEM: Transmission electron microscopy; ThT: Thioflavin T; WT: Wild-type.
