# Supplementary material for: Site-Specific Aspartic Acid d-Isomerization in Tau R2 and R3 Peptide Seeds Attenuates Seed-Induced Fibril Formation of Full-Length Tau
Source: Biomolecules. 2026 Jan 13;16(1):143. doi: 10.3390/biom16010143 (PMC12839334; doi:10.3390/biom16010143)
Supplement: Supplementary file 1 [file biomolecules-16-00143-s001.zip › biomolecules-4056477_suppl.pdf]

Supplementary figure legends

Figure S1. Individual curves corresponding to Figure 2A and 2B.

Each curve represents the mean value of an independent experiment performed in triplicate.

Figure S2–S8. Individual curve fits for WT tau (1N4R) fibril formation for Figure 2C–2F.

Curve fitting was performed on individual replicates from four independent experiments (three replicates each). The notation "X-Y" denotes replicate Y of experiment X (e.g., 1-1). Fitted curves are shown in blue, and original normalized fluorescence data in gray.

Figure S9. Individual curves corresponding to Figure 4A and 4B.

Each curve represents the mean value of an independent experiment performed in triplicate.

Figure S10–S16. Individual curve fits for P301S tau (1N4R) fibril formation for Figure 4C–4F.

Curve fitting was performed on individual replicates from four independent experiments (three replicates each). The notation "X-Y" denotes replicate Y of experiment X (e.g., 1-1). Fitted curves are shown in blue, and original normalized fluorescence data in gray.

Figure S17. Original uncropped images for Figure 5B.

Figure S18. Individual channel images for Figure 5C.

Figure S19. Original uncropped images for Figure 5D.

Figure S1

Tau (1N4R) WT + seed

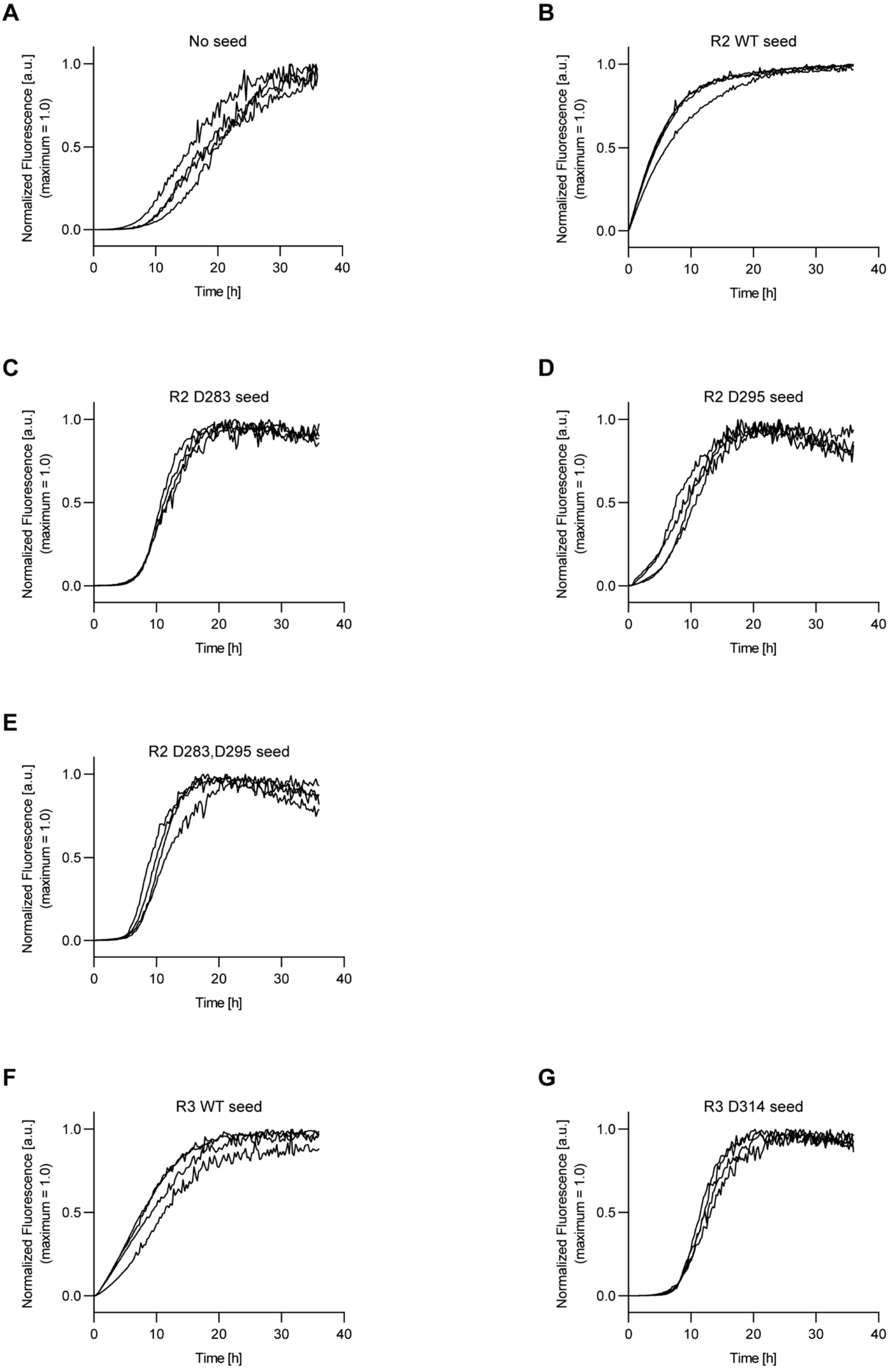

Figure S2

Tau (1N4R) WT (no seed)

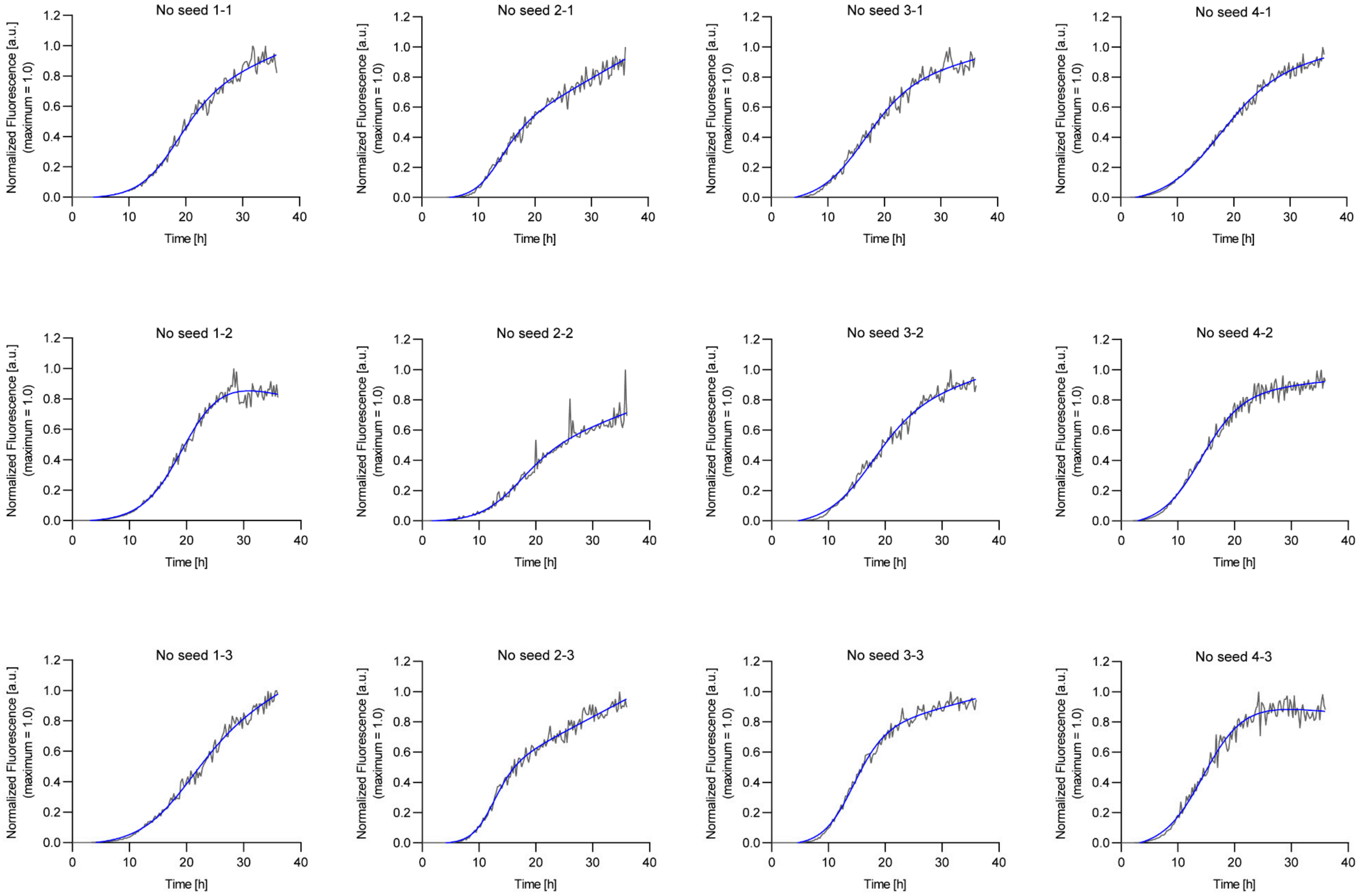

Figure S3

Tau (1N4R) WT + R2 WT seed

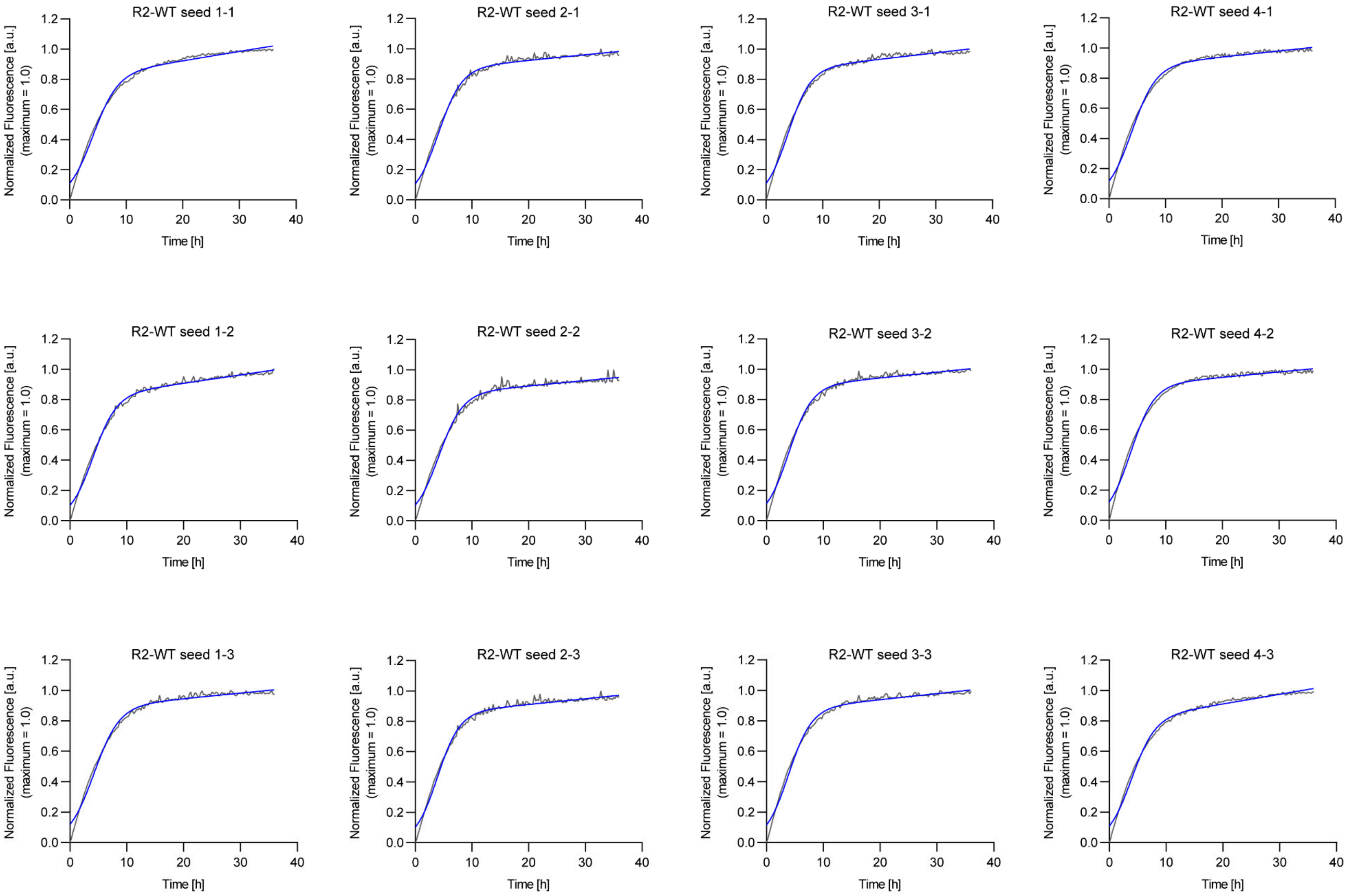

Figure S4

<u>Tau (1N4R) WT + R2-D283 seed</u>

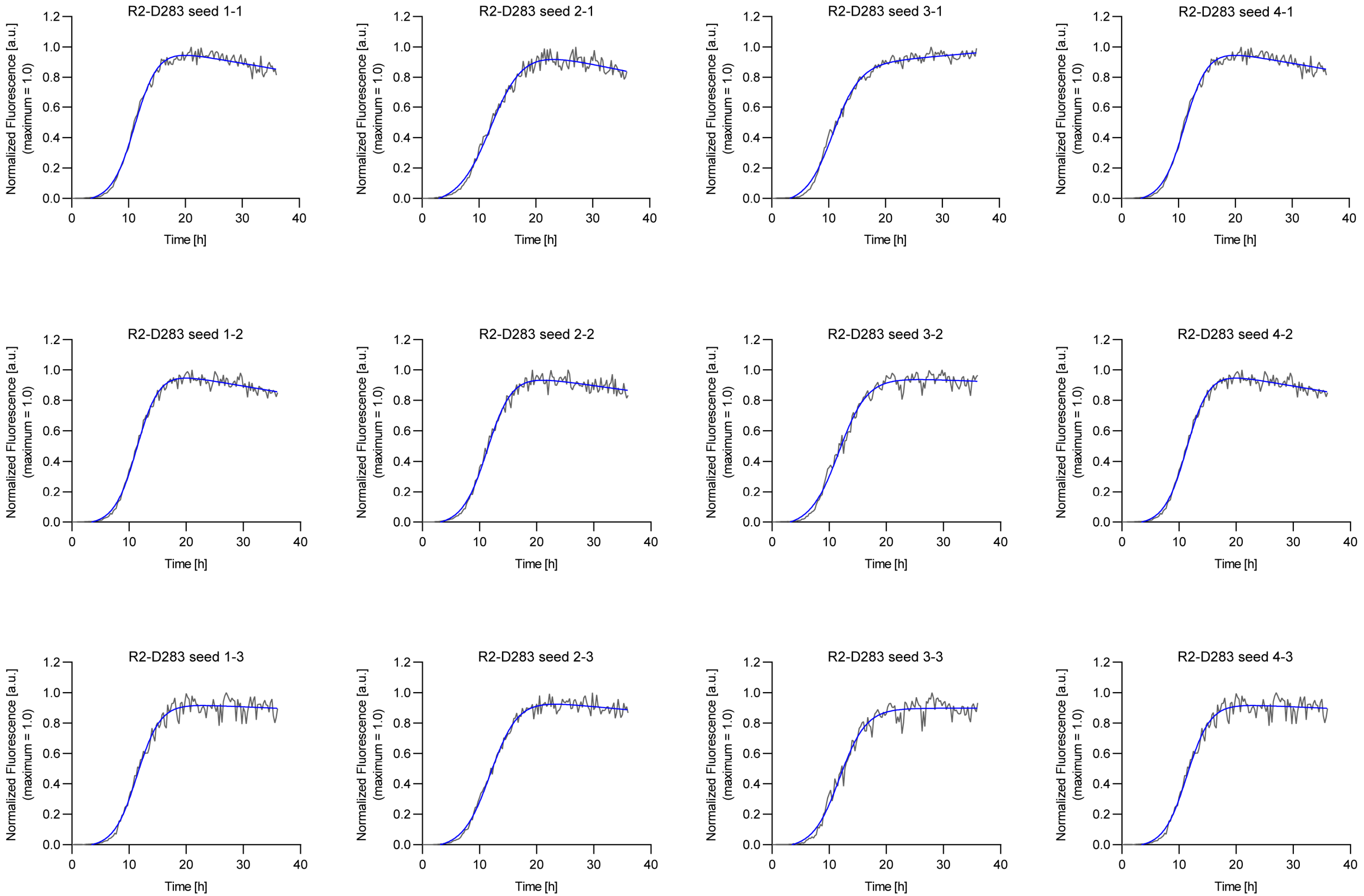

Figure S5

Tau (1N4R) WT + R2 D295 seed

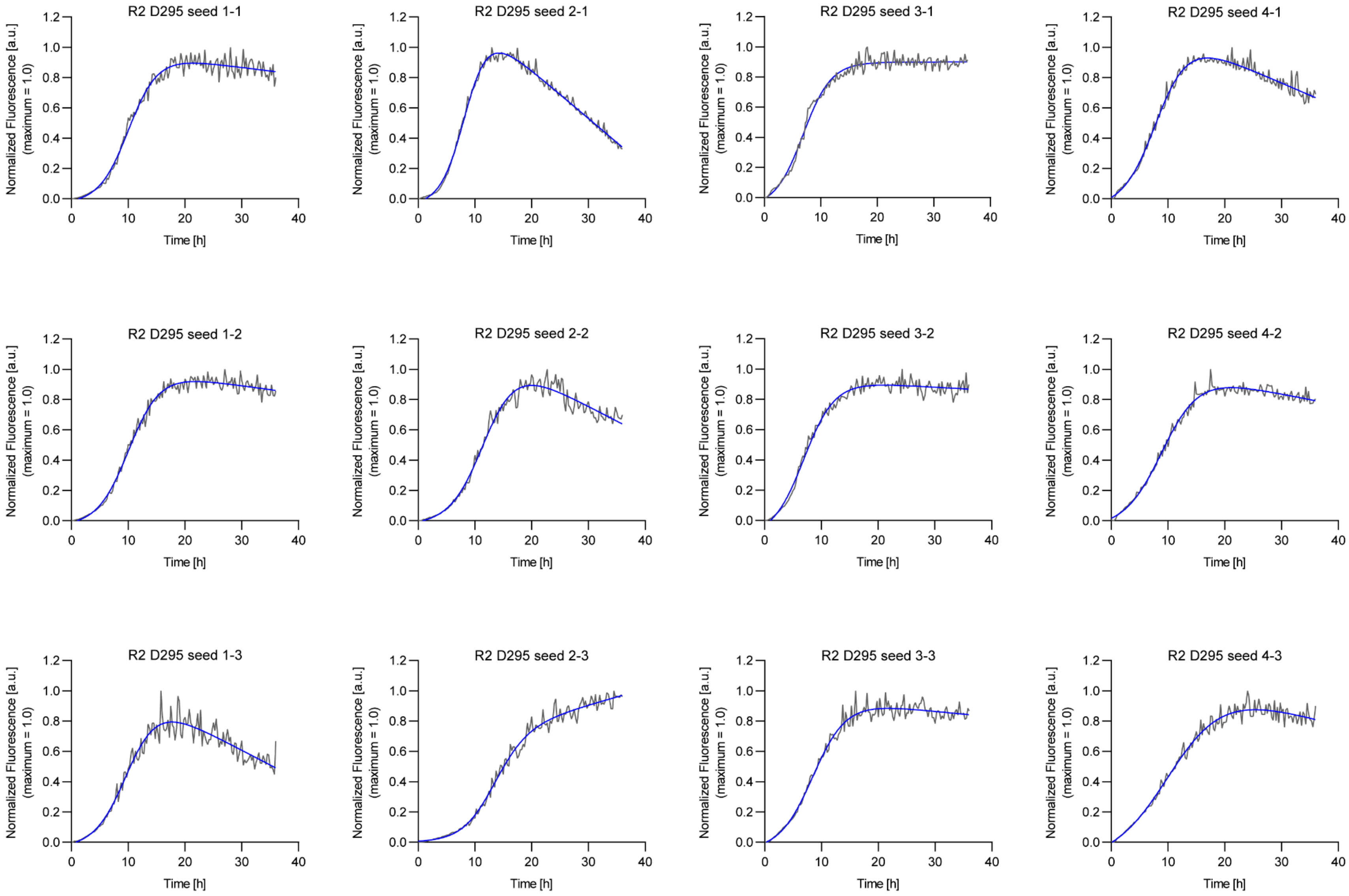

Figure S6

Tau (1N4R) WT + R2 D283,D295 seed

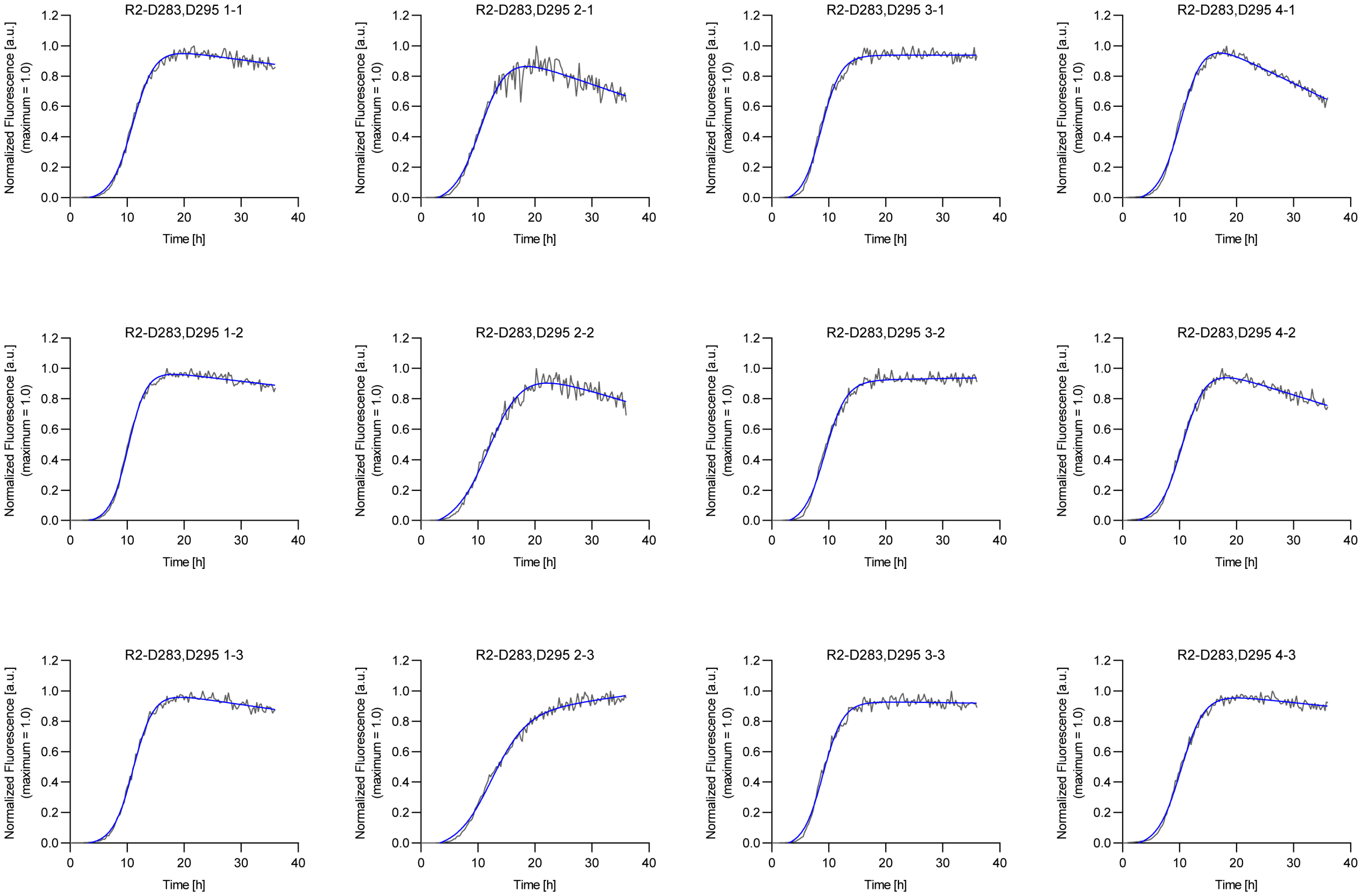

Figure S7

Tau (1N4R) WT + R3 WT seed

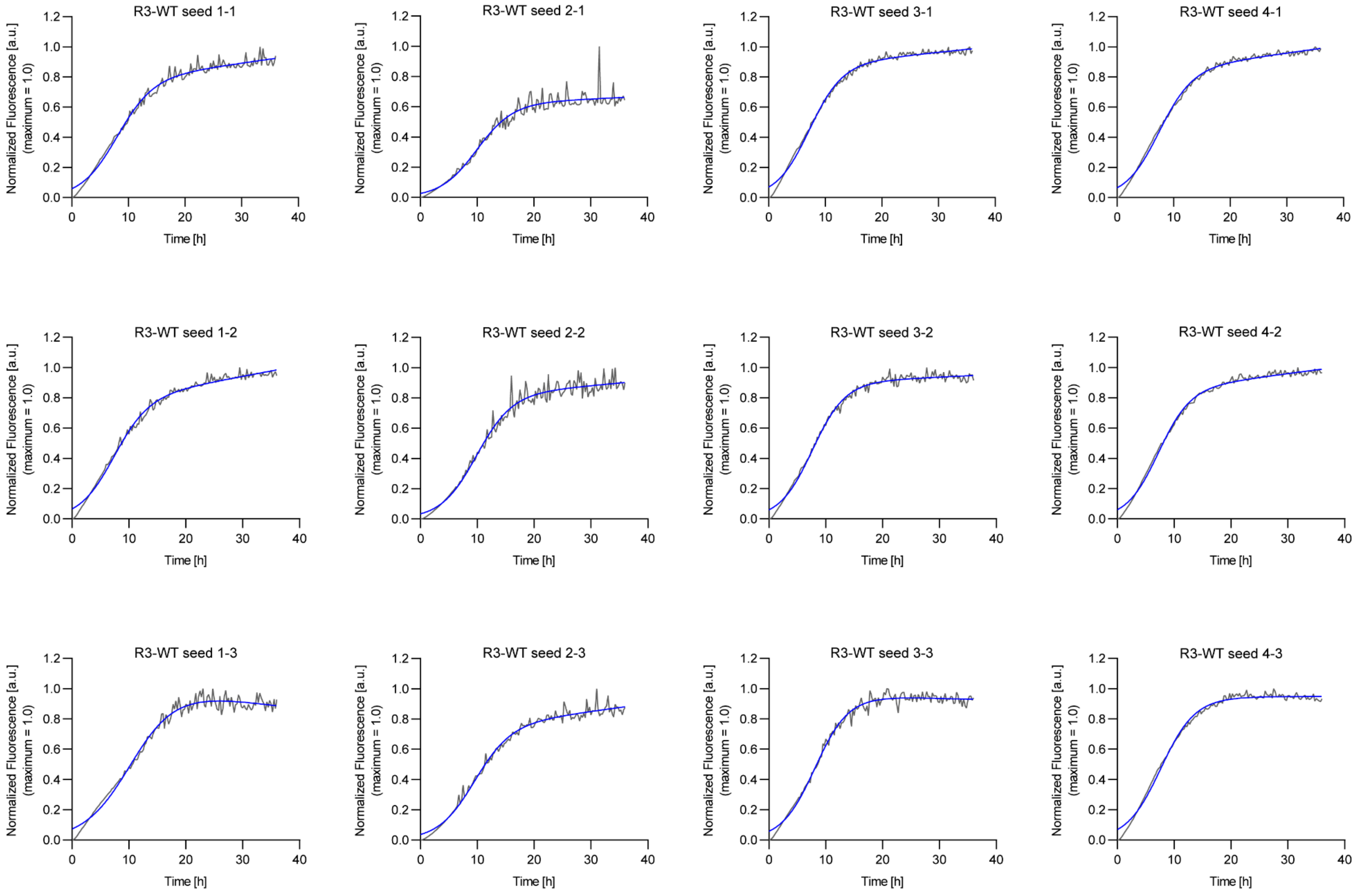

Figure S8

Tau (1N4R) WT + R3 D314 seed

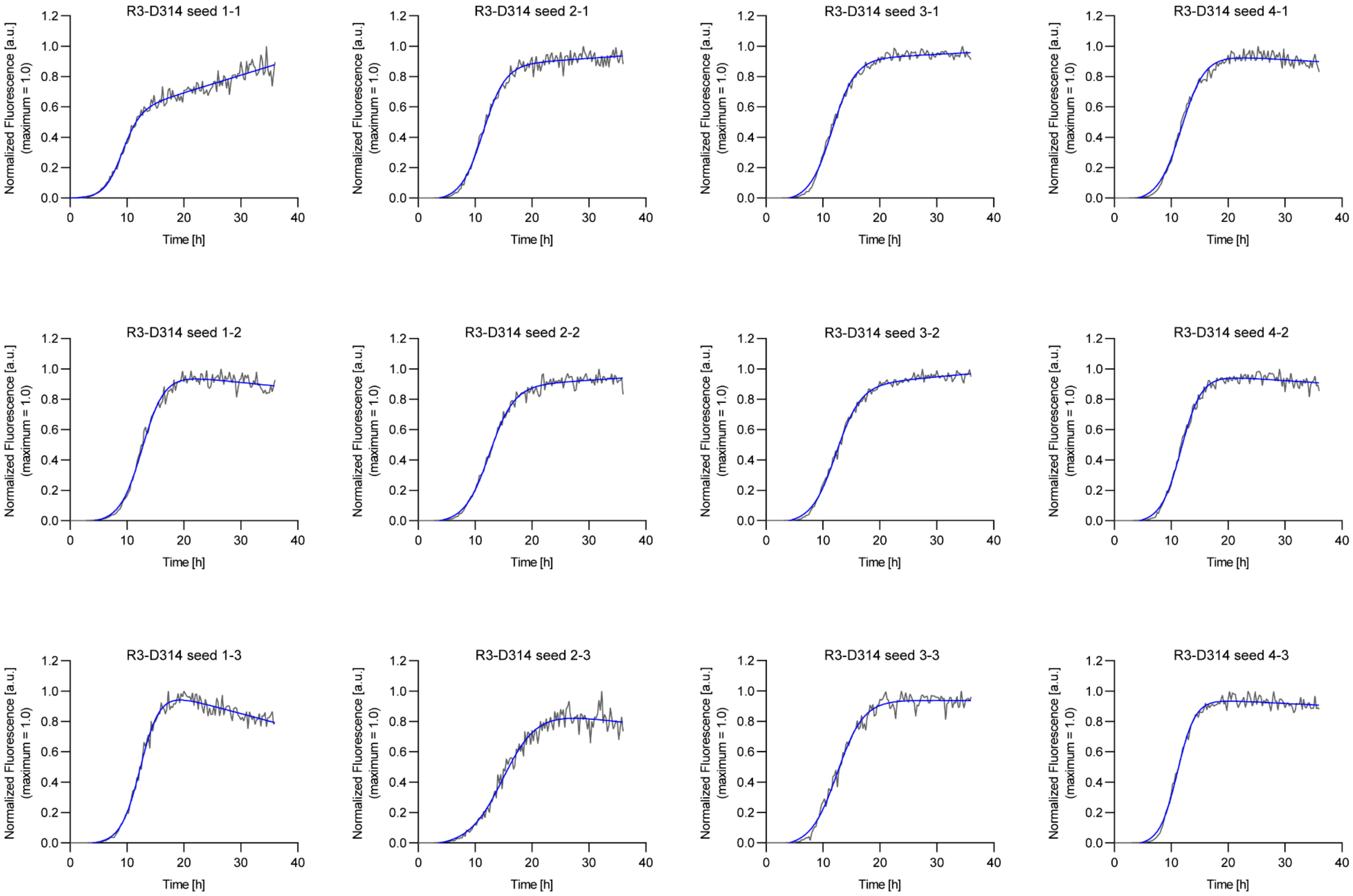

Figure S9

<u>Tau (1N4R) P301S + seed</u>

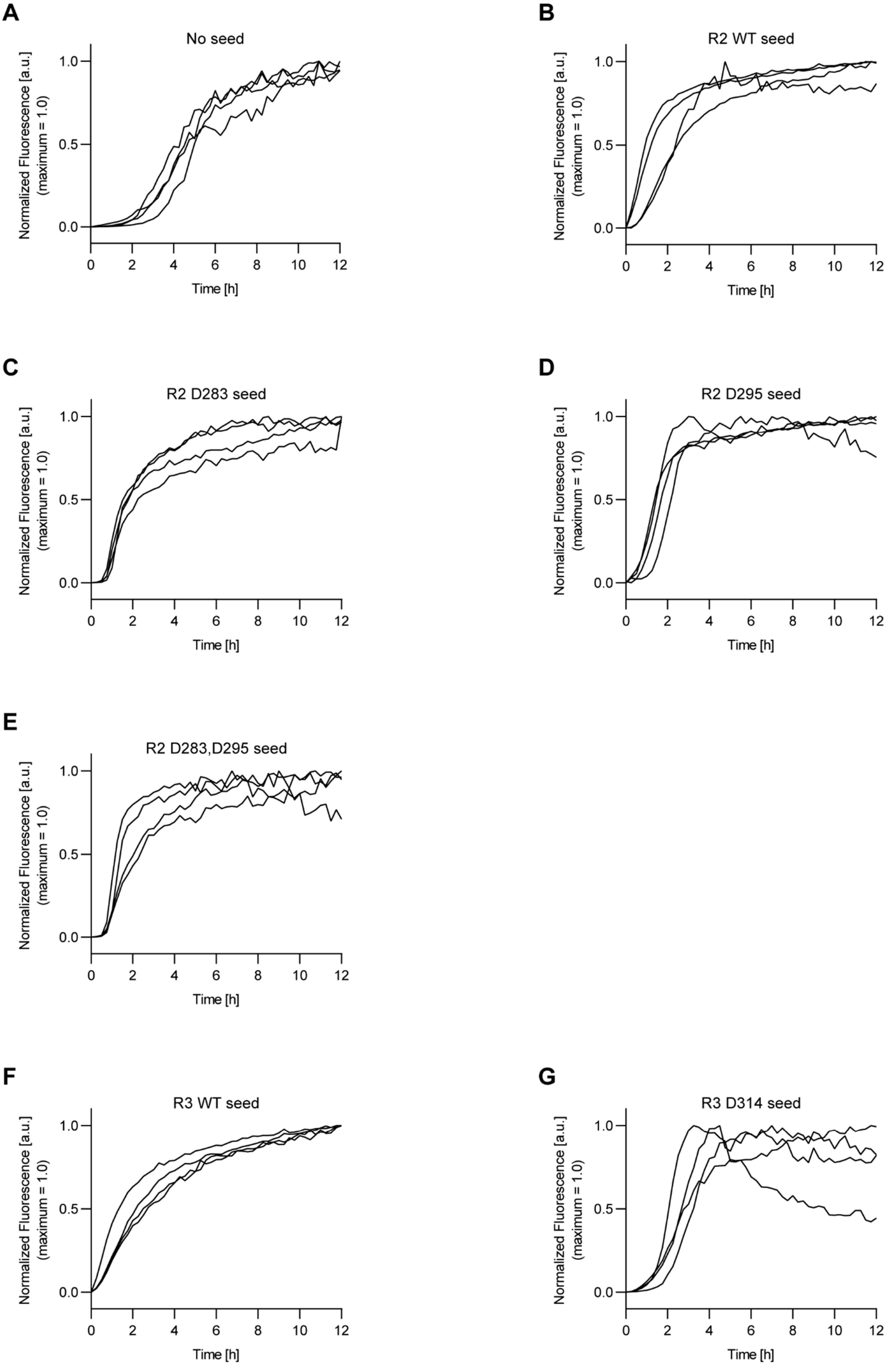

Figure S10

<u>Tau (1N4R) P301S (no seed)</u>

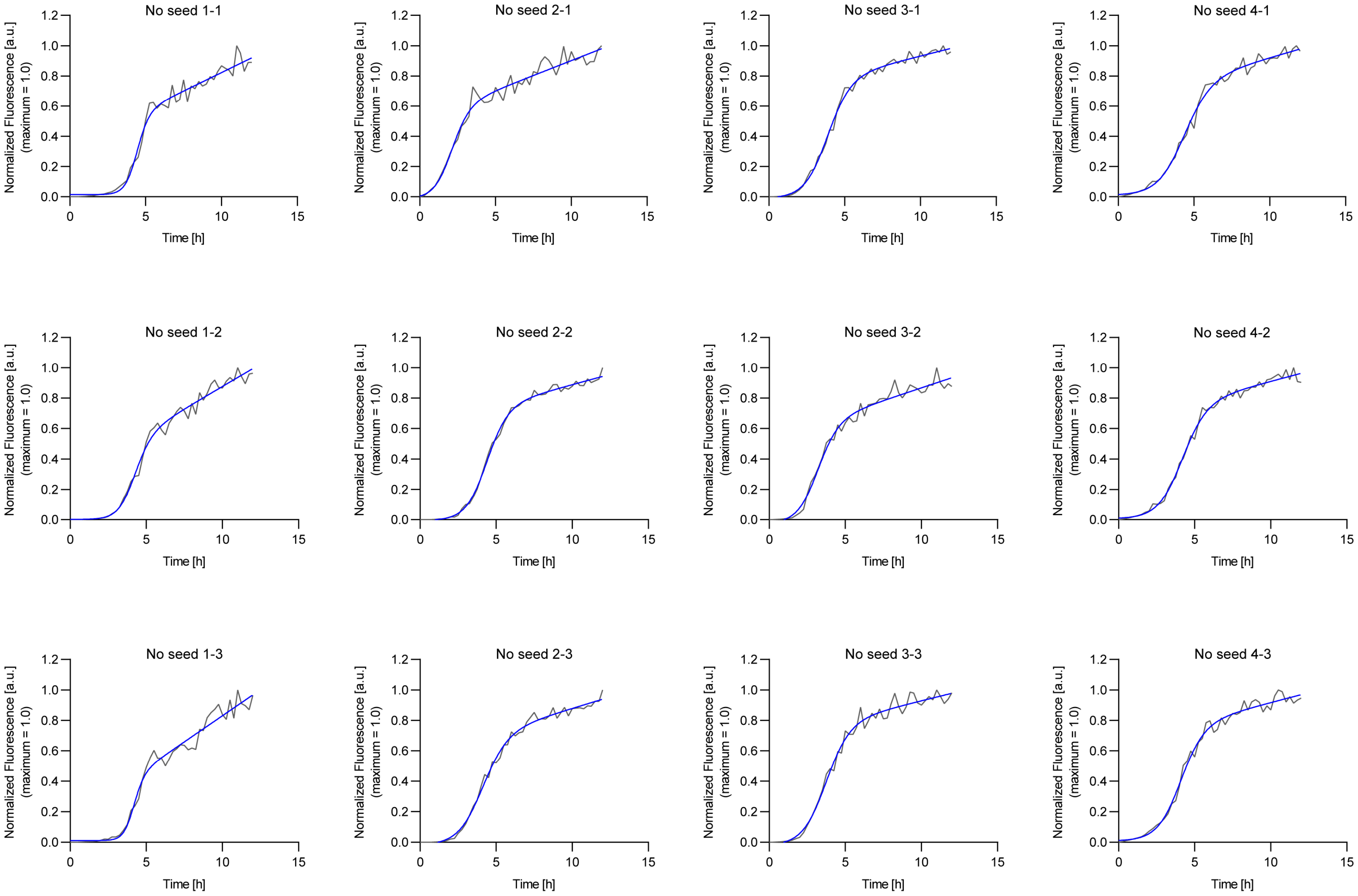

Figure S11

Tau (1N4R) P301S + R2 WT seed

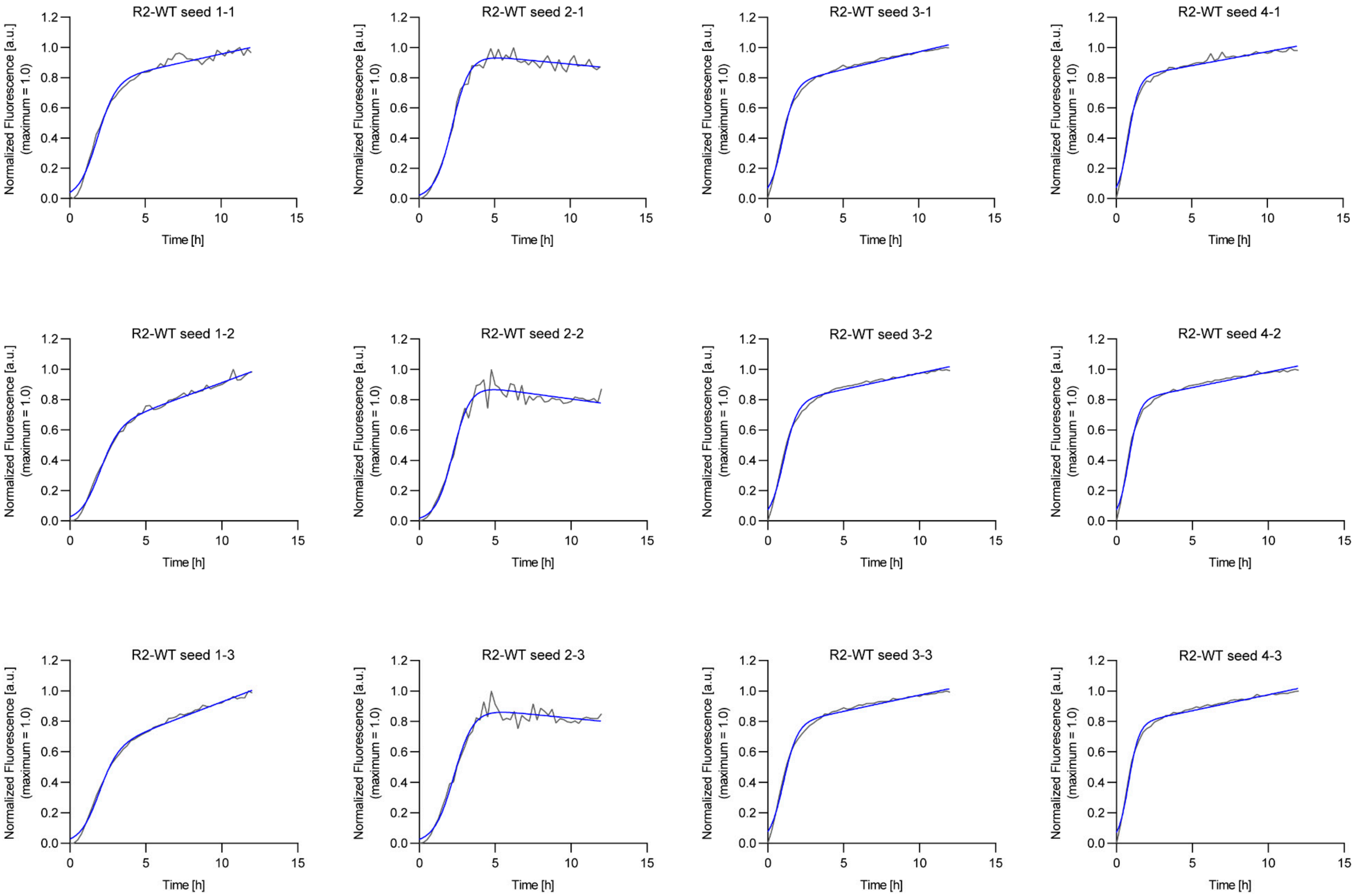

Figure S12

Tau (1N4R) P301S + R2 D283 seed

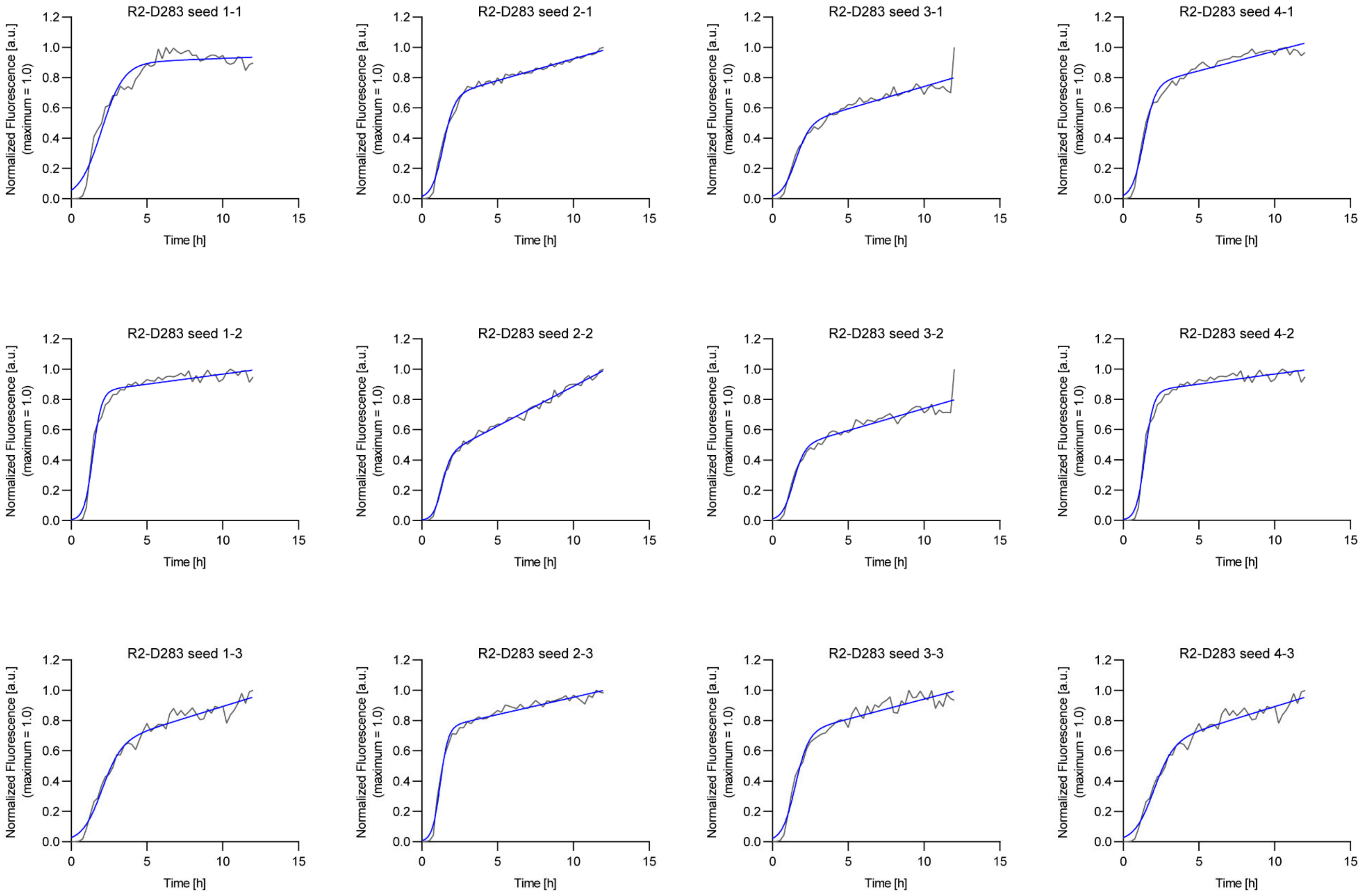

Figure S13

Tau (1N4R) P301S + R2 D295 seed

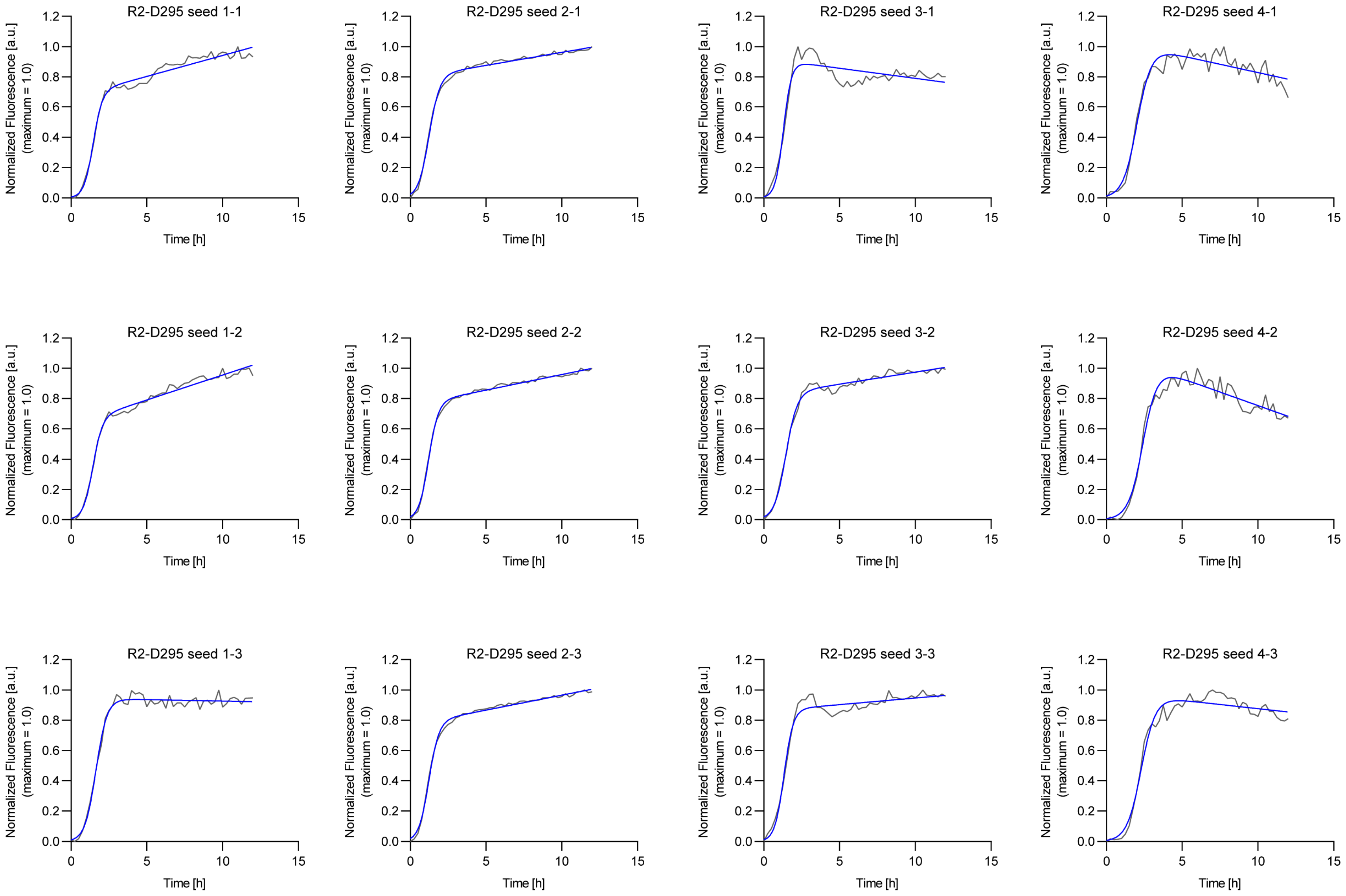

Figure S14

Tau (1N4R) P301S + R2 D283,D295 seed

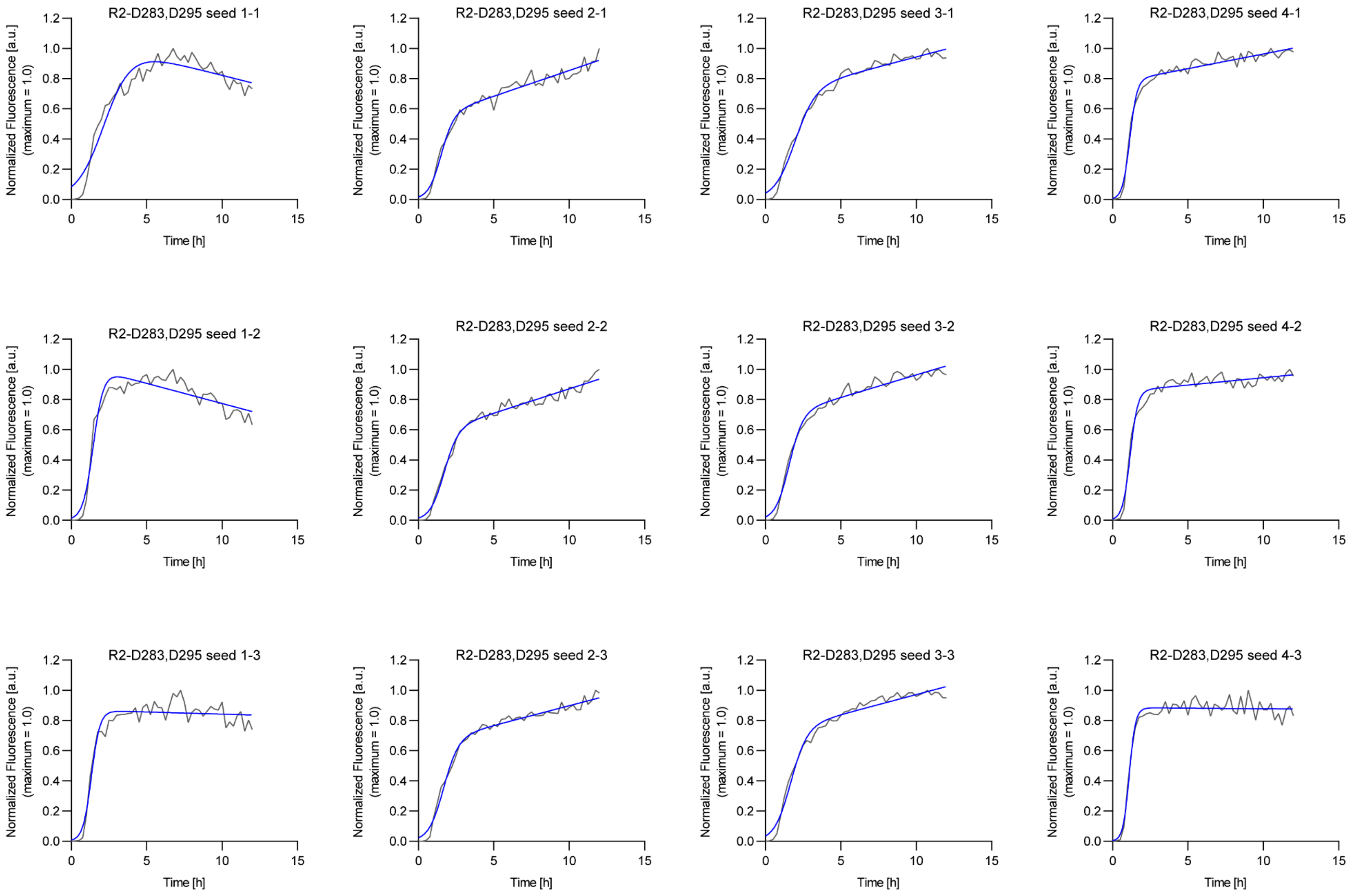

Figure S15

Tau (1N4R) P301S + R3 WT seed

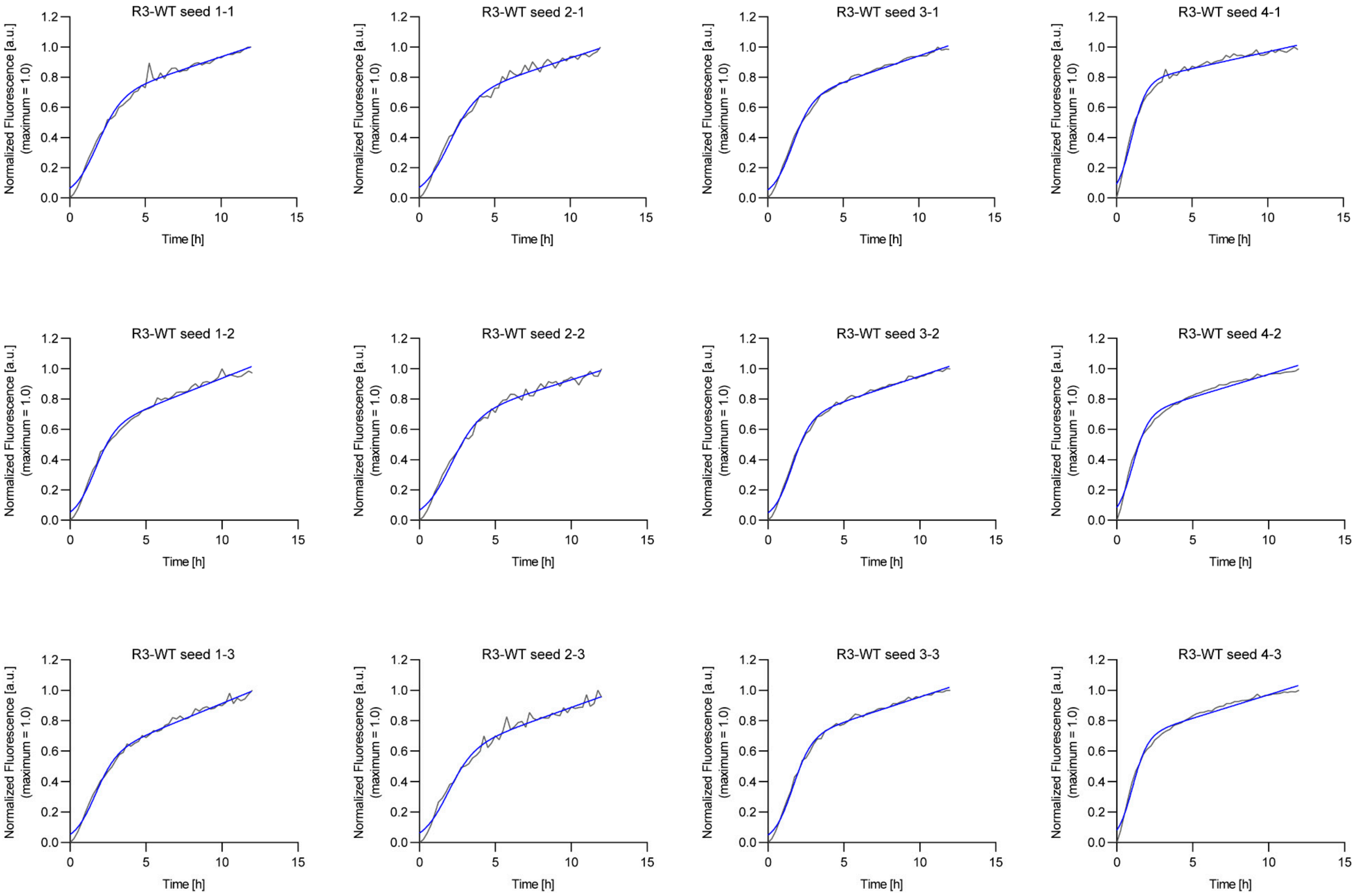

Figure S16

Tau (1N4R) P301S + R3 D314 seed

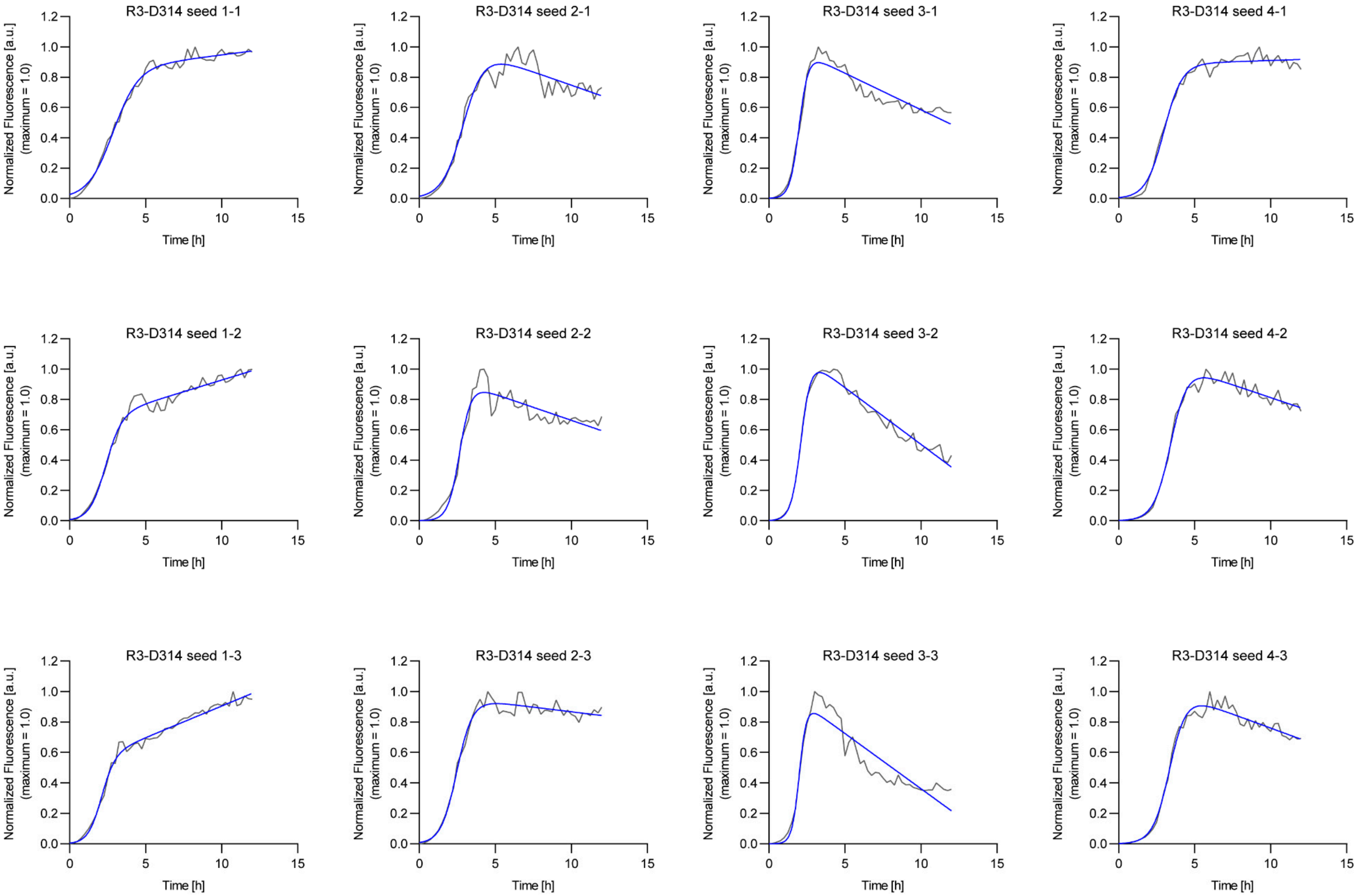

Figure S17

Original uncropped images for Figure 5B

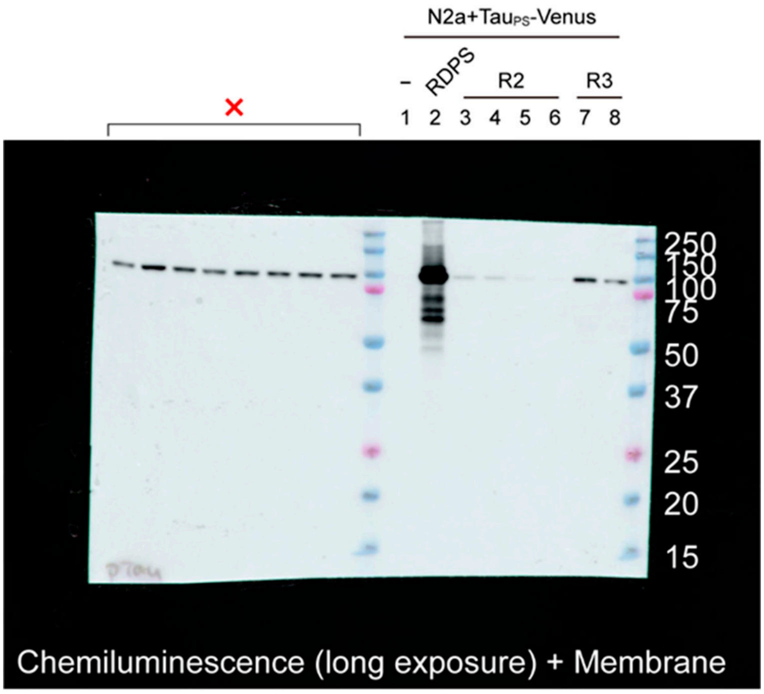

pS202/pT205 tau

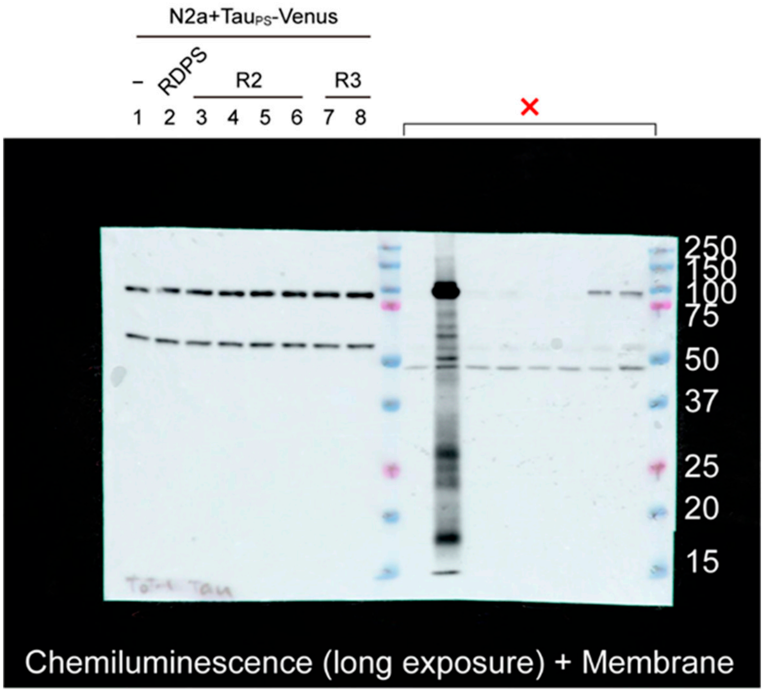

Total tau

Figure S18

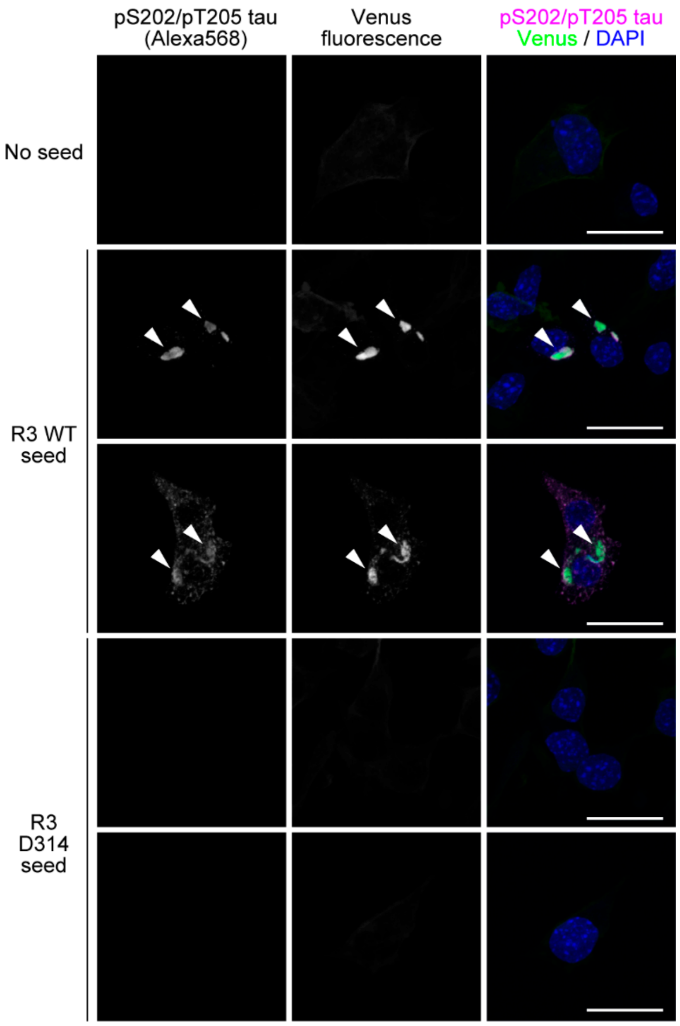
pS202/pT205 tau
(Alexa568)
Venus
fluorescence
pS202/pT205 tau
Venus / DAPI
No seed
R3 WT
seed
R3
D314
seed

Figure S19

Original uncropped images for Figure 5D

Experiment 1

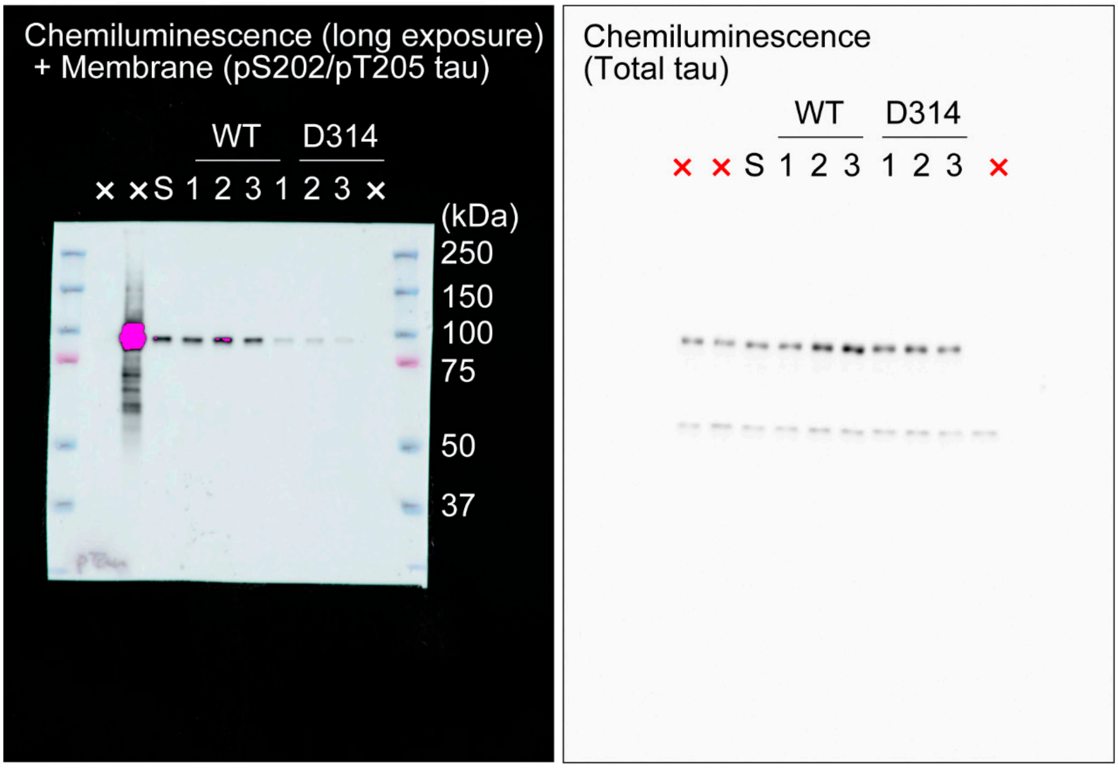

The sample "S" is a common reference sample for band intensity normalization.

Original uncropped images for Figure 5D

Experiment 2 and 3
(Samples were processed independently on different days for each experiment.)

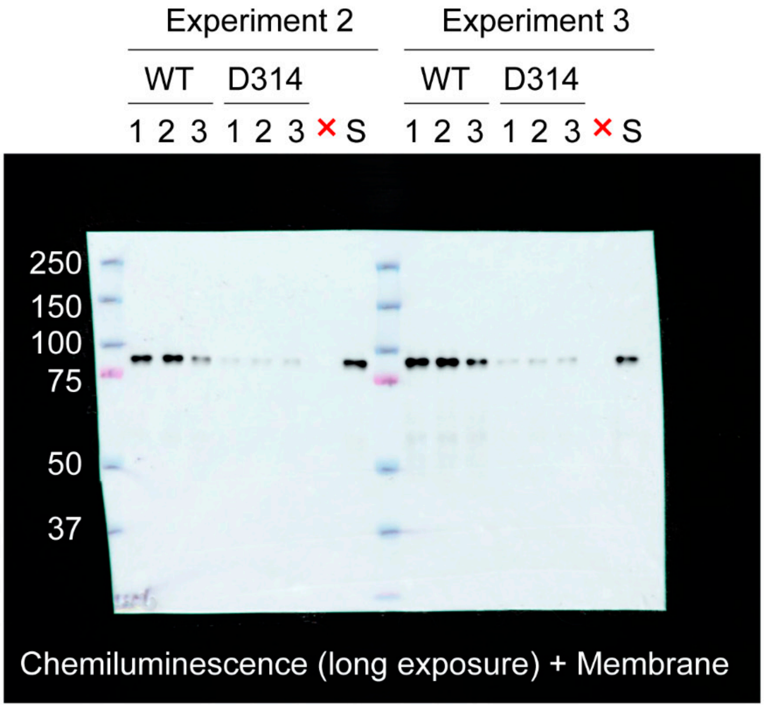

pS202/pT205 tau

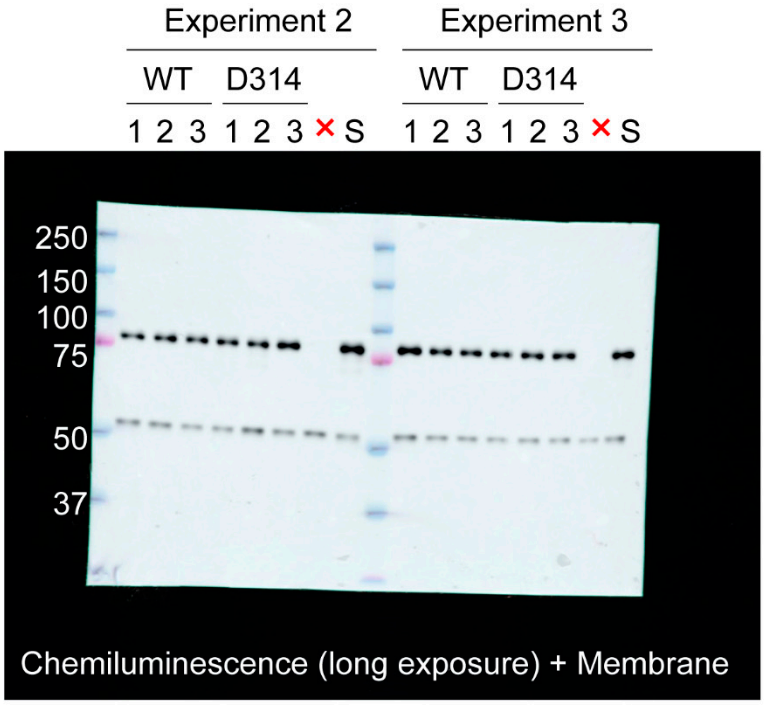

Total tau

The sample “S” is a common reference sample for band intensity normalization.
